# Lysosomal Targeting Enhancement by Conjugation of Glycopeptides Containing Mannose-6-phosphate Glycans Derived from Glyco-engineered Yeast

**DOI:** 10.1038/s41598-018-26913-4

**Published:** 2018-06-07

**Authors:** Ji-Yeon Kang, Keun Koo Shin, Ha Hyung Kim, Jeong-Ki Min, Eun Sun Ji, Jin Young Kim, Ohsuk Kwon, Doo-Byoung Oh

**Affiliations:** 1 Synthetic Biology and Bioengineering Research Center, Korea Research Institute of Bioscience and Biotechnology (KRIBB), 125 Gwahakro, Yuseong-gu, Daejeon, 34141 Korea; 2 Biotherapeutics and Glycomics Laboratory, College of Pharmacy, Chung-Ang University, 84 Heukseok-ro, Dongjak-gu, Seoul, 06944 Korea; 3 Biotherapeutics Translational Research Center, Korea Research Institute of Bioscience and Biotechnology (KRIBB), 125 Gwahakro, Yuseong-gu, Daejeon 34141 Korea; 4 Department of Biomolecular Science, University of Science and Technology (UST), Daejeon, 34113 Korea; 5 Biomedical Omics Research Center, Korea Basic Science Institute, Ochang, Chungbuk 28119 Korea; 6 Department of Biosystems and Bioengineering, University of Science and Technology (UST), Daejeon, 34113 Korea

## Abstract

Many therapeutic enzymes for lysosomal storage diseases require a high content of mannose-6-phosphate (M6P) glycan, which is important for cellular uptake and lysosomal targeting. We constructed glyco-engineered yeast harboring a high content of mannosylphosphorylated glycans, which can be converted to M6P glycans by uncapping of the outer mannose residue. In this study, the cell wall of this yeast was employed as a natural M6P glycan source for conjugation to therapeutic enzymes. The extracted cell wall mannoproteins were digested by pronase to generate short glycopeptides, which were further elaborated by uncapping and α(1,2)-mannosidase digestion steps. The resulting glycopeptides containing M6P glycans (M6PgPs) showed proper cellular uptake and lysosome targeting. The purified M6PgPs were successfully conjugated to a recombinant acid α-glucosidase (rGAA), used for the treatment of Pompe disease, by two-step reactions using two hetero-bifunctional crosslinkers. First, rGAA and M6PgPs were modified with crosslinkers containing azide and dibenzocyclooctyne, respectively. In the second reaction using copper-free click chemistry, the azide-functionalized rGAA was conjugated with dibenzocyclooctyne-functionalized M6PgPs without the loss of enzyme activity. The M6PgP-conjugated rGAA had a 16-fold higher content of M6P glycan than rGAA, which resulted in greatly increased cellular uptake and efficient digestion of glycogen accumulated in Pompe disease patient fibroblasts.

## Introduction

Mannose-6-phosphate (M6P) glycan is a crucial signal for the trafficking of lysosomal enzymes to lysosomes. Most lysosomal enzymes are glycoproteins decorated with high-mannose type glycans as well as complex type glycans. These high-mannose type glycans are often modified with M6P in the Golgi through the action of two enzymes^1,2^: GlcNAc-1-phosphotransferase adds GlcNAc-1-phosphate to the C6 hydroxyl group of specific mannose residues and, then, uncovering enzyme removes the outer GlcNAc to leave a phosphate group linked to the mannose residue (phosphate-6-*O*-mannose), which is M6P. The M6P glycans of lysosomal enzymes are recognized by M6P receptors (MPRs) and the resulting enzyme-MPR complex is delivered to early endosomes. As the pH decreases during endosome maturation, lysosomal enzymes are released from MPRs and finally go to the lysosome through a fusion of late endosome to the lysosome. Although some enzymes escaping from MPR binding are secreted to extracellular space, they can be recaptured by MPRs and subsequently targeted to the lysosome through receptor-mediated endocytosis. Many therapeutic enzymes, which are used to treat lysosomal storage disease patients, exploit this MPR-based recapturing pathway for cellular uptake and lysosomal targeting.

Lysosomal storage diseases are caused by a lysosomal enzyme deficiency, which results in abnormal accumulation of undigested molecules^2,3^. Because this induces cellular malfunctions leading to multiple tissue and organ failures, patients develop various clinical symptoms in childhood and often end up with early death. Currently, 10 therapeutic enzymes are approved for treatment of seven lysosomal storage diseases^2,4^. Except for three enzymes for Gaucher disease, the other seven enzymes use the M6P glycan-MPR pathway for proper lysosomal targeting. Because M6P glycan content is a key factor for lysosomal targeting and therapeutic efficacy of these enzymes^5,6^, glyco-engineering strategies have been developed to increase the M6P glycan content^2^. Especially, recombinant acid α-glucosidase (rGAA; alglucosidase alfa; Myozyme) for Pompe disease has been a prevalent target because the high dose of rGAA can only partially reduce the glycogen level in skeletal muscle, owing to its low content (<5%) of M6P glycan^7,8^. Among many approaches focused on rGAA^5,9–13^, Genzyme researchers reported that conjugation of synthetic M6P glycans to periodate-oxidized sialic acids of rGAA was very effective and resulted in a greater potency in Pompe model mice^12^. Although the M6P glycan conjugation strategy showed promise for improved potency, chemical synthesis of M6P glycans is a laborious multistep process because repetitive protection, deprotection, and extensive purification steps are indispensable^14,15^. Natural M6P glycans isolated from another therapeutic enzyme GLA (Agalsidase beta; Fabrazyme) have been used to prove the concept that M6P glycan conjugation increased the potency of rGAA^9^. However, because the amount of M6P glycans obtained from lysosomal enzymes was highly limited and its production by using mammalian cell culture was not economical, this approach did not lead to further development.

Yeast glyco-engineering approaches have been highlighted because they have enabled the production of therapeutic enzymes containing a high level of M6P glycans^7,16–18^. Although wild-type yeasts do not have M6P glycans, their high-mannose type glycans often contain a mannosylphosphorylated mannose structure, which can be converted to M6P glycans through uncapping of the outer mannose^7,16–18^. Especially, Tiels *et al*. reported that rGAA produced in glyco-engineered *Yarrowia lipolytica* was modified with a high content of M6P glycans, which displayed a greatly increased potency in Pompe mice^7^. In our previous study, we showed that *YlMPO1* overexpression in glyco-engineered *Saccahromyces cerevisiae* can lead to the production of recombinant protein containing a high amount (>80%) of M6P glycans^18^. Unfortunately, secretory expression levels of therapeutic enzymes from the glyco-engineered *S. cerevisiae* were very low. Instead of employing it directly for enzyme production, we thought that this yeast could be used as a natural source of M6P glycans for conjugation to enzymes, which would be much more economical than M6P glycans obtained from lysosomal enzymes.

In this study, we employed glycopeptides containing M6P glycans (M6PgPs) prepared from the cell wall mannoproteins of glyco-engineered *S. cerevisiae* because M6PgPs contain N-terminal α-amino group useful for chemical conjugation. The M6PgPs were successfully linked to a therapeutic enzyme, rGAA, through a two-step conjugation reaction involving copper-free click chemistry. The resulting M6PgP-conjugated rGAA showed greatly increased cellular uptake and lysosomal targeting.

## Results

### Preparation of M6PgPs from cell wall mannoproteins of glyco-engineered *S. cerevisiae*

In a previous study, our group constructed a glyco-engineered *S. cerevisiae* strain overexpressing the *YlMPO1* gene (*och1Δmnn1Δ*/*YlMPO1*); the *OCH1* and *MNN1* genes were disrupted to block yeast-specific hyper-mannosylation and immunogenic α(1,3)-mannosylation while the *YlMPO1* gene was introduced to increase mannosylphosphorylation^18^. Mannosylphosphorylated mannose residues (mannose-1-phosphate-6-*O*-mannose) can be converted to M6P structures (phosphate-6-*O*-mannose) by uncapping of the outer mannoses using mild acid hydrolysis (MAH) or uncapping enzyme^7,19^. We thought that cell wall mannoproteins, a major component of yeast cell wall, would be a good source for M6P glycans because they can be easily obtained from the harvested cells^18,20^. The M6PgPs were prepared from cell wall mannoproteins through pronase digestion and subsequent uncapping, trimming, and purification processes.

**Figure 1:**
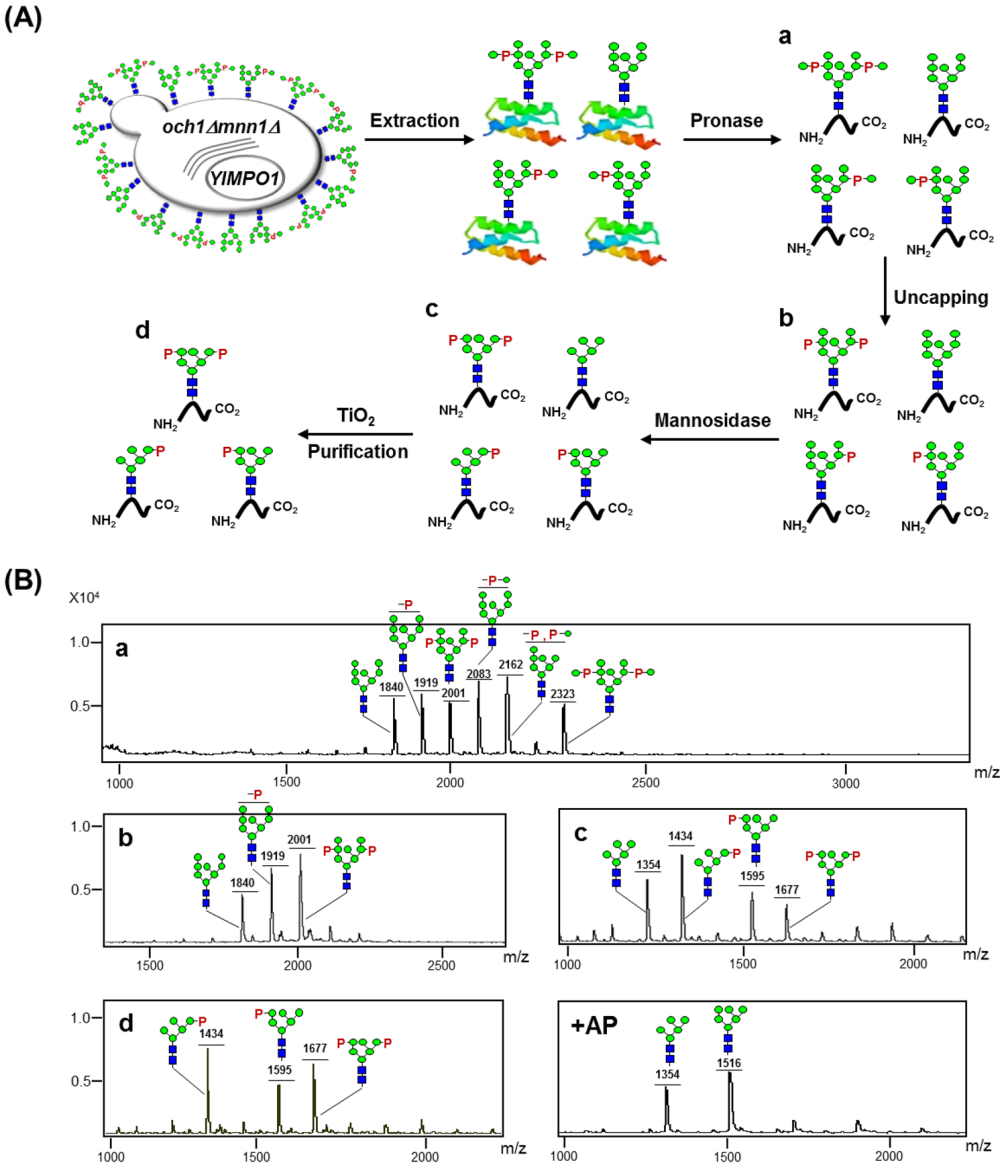
Preparation of M6PgPs from the glyco-engineered *S. cerevisiae*. (**A**) Schematic representation of M6PgP preparation procedure. Cell wall mannoproteins of the glyco-engineered yeast were digested with pronase, which generated short glycopeptides (a). The glycopeptides were uncapped for generation of M6P glycans by MAH (b) and then digested with α(1,2)-mannosidase (c). Finally, the resulting M6PgPs were purified by using TiO_2_ column (d). (**B**) *N*-glycans of the glycopeptides after each preparation step (a–d) were analyzed in a linear negative ion mode after 2-AA labeling (which adds 121 Da) by using MALDI-TOF mass spectrometry^22^. For confirmation of phosphate group existence, alkaline phosphatase was treated (+AP). Monosaccharide symbols follow the SNFG (Symbol Nomenclature for Glycans) system, details of which are found at NCBI^40^: green circle, mannose; blue square, GlcNAc; P, phosphate.

The M6PgP preparation procedure is summarized in Fig. 1A. After culturing *S. cerevisiae och1Δmnn1Δ*/*YlMPO1* strain, cell wall mannoproteins were isolated from the harvested cells by hot citrate buffer extraction and subsequent ethanol precipitation. They were digested with pronase, which generated very short glycopeptides together with free amino acids^21^. The glycopeptides were efficiently purified on a graphitized carbon column due to the glycan moiety being much larger than the peptide part. Their glycans were released by PNGase F treatment and analyzed by matrix-assisted laser desorption ionization time-of-flight (MALDI-TOF) mass spectrometry (Fig. 1B-a), which showed four types of glycans: neutral (Man_8_GlcNAc_2_), mannosylphosphorylated [Man-P-Man_8_GlcNAc_2_ and (Man-P)_2_-Man_8_GlcNAc_2_], phosphorylated (P-Man_8_GlcNAc_2_ and P_2_-Man_8_GlcNAc_2_), and mannosylphosphorylated and phosphorylated (Man-P-/P-Man_8_GlcNAc_2_) glycans. Here, the phosphorylated glycan structures were generated from the unexpected uncapping reaction induced by the acidic matrix preparation step for MALDI-TOF mass spectrometry analysis, which was previously reported^22^. After MAH for uncapping, all mannosylphosphorylated mannose moieties were converted to M6P structures, which resulted in a simple glycan profile with neutral (Man_8_GlcNAc_2_), mono-phosphorylated (P-Man_8_GlcNAc_2_), and bi-phosphorylated (P_2_-Man_8_GlcNAc_2_) glycans (Fig. 1B-b). Through α(1,2)-mannosidase treatment, neutral and bi-phosphorylated glycans were trimmed to Man_5_GlcNAc_2_ and P_2_-Man_6_GlcNAc_2_ glycans. On the other hand, the mono-phosphorylated glycan was converted to P-Man_5_GlcNAc_2_ and P-Man_6_GlcNAc_2_ glycans depending on the phosphorylation site (Fig. 1B-c). Here, P-Man_6_GlcNAc_2_ glycan can be created only if the phosphorylation site is located on the penultimate α(1,2)-mannose residue of α(1,3)-arm because α(1,2)-mannosidase does not remove the phosphorylated mannose (Supplementary Fig. S1A). In contrast, all α(1,2)-mannoses can be digested to generate P-Man_5_GlcNAc_2_ glycan when the penultimate α(1,6)-mannose residue of α(1,6)-arm is phosphorylated (Supplementary Fig. S1B). The M6PgPs were purified from the neutral glycopeptides by using titanium dioxide (TiO_2_) chromatography (Fig. 1A-d), which was reported to purify negatively charged peptides such as phosphorylated peptides^23^ and sialylated glycopeptides^24^. The M6PgPs were selectively purified because the negative charges of phosphates (Fig. 1B-d) and existence of phosphate groups in the purified M6PgPs was confirmed by alkaline phosphatase treatment (Fig. 1B-e).

The glycopeptides purified by TiO_2_ chromatography were also directly analyzed to know the lengths of peptides and amino acid sequences by using the liquid chromatography tandem-mass (LC-MS/MS) spectrometry (Supplementary Figs S2–4). Because pronase digestion generated very short peptides without specific cleavage sites, we could not obtain the amino acid sequence information. Instead, the molecular weights of peptide parts could be determined from the Y0 ion (peptide + H^+^) (Table 1). The Y0 ion masses of 23 identified glycopeptides ranged from 280.06 to 938.51. The average molecular weight of peptide parts was calculated to be 564.95 Da (Supplementary Fig. S3), suggesting that the average peptide length would be ~5.1 amino acids upon considering the average molecular weight of amino acids (110 Da). The 14 glycopeptides were found to contain P-Man_5_GlcNAc_2_ glycan, which is the strongest peak in the glycan profile analysis by using MALDI-TOF mass spectrometry (Fig. 1B-d). Two glycopeptides carried P-Man_6_GlcNAc_2_ glycan while the glycopeptide containing P_2_-Man_6_GlcNAc_2_ glycan was not found in proteomics analysis (Table 1) although the P_2_-Man_6_GlcNAc_2_ glycan peak is stronger than the P-Man_6_GlcNAc_2_ peak in the glycan profile analysis (Fig. 1B-d). Notably, five glycopeptides containing non-phosphorylated high-mannose type glycans were identified (Table 1). Minor amounts of six glycopeptides containing *O*-linked mannoses or phosphomannoses were also found in the LC-MS/MS analysis (Supplementary Table S1). Here, only two Y0 ion masses (903.56 and 793.52) existed in the six glycopeptides, suggesting that two peptides were attached with the different numbers of mannoses (Man_5–7_). Notably, these chain lengths appeared to be considerably long, considering that *S. cerevisiae* usually forms *O*-linked Man_1–6_ in length^25^. It was suspected that the only glycopeptides containing long chains of *O*-linked mannoses were retrieved because the ones harboring short chains were lost during the graphitized carbon column purification step.

**Table 1:** List of the glycopeptides identified by HCD and CID MS/MS spectra.

Peak No	RT (min)	m/z	Charge State	Experimental MW	Identified glycan	MW of glycan	Y0 ion^*^	Y1 ion^*^	Calculated MW
1^†^	14.94	1392.54	2	2783.08	HexNAc(2)Hex(9)^#^	1864.45	919.60	1122.59	2783.05
2	17.50	788.33	2	1574.65	HexNAc(2)Hex(5)	1216.42	359.25	562.31	1574.67
3	18.67	1089.89	2	2177.78	HexNAc(2)Hex(7)P	1620.49	558.30	761.30	2177.79
4^†^	18.89	839.82	2	1677.65	HexNAc(2)Hex(5)	1216.42	462.22	665.28	1677.64
5^†^	20.40	788.76	2	1575.52	HexNAc(2)Hex(5)P	1296.40	280.06	483.20	1575.46
6^†^	20.62	800.28	2	1598.56	HexNAc(2)Hex(5)P	1296.40	303.12	506.22	1598.52
7	20.71	850.80	2	1699.60	HexNAc(2)Hex(5)P	1296.40	404.21	607.19	1699.61
8	21.16	815.29	2	1628.57	HexNAc(2)Hex(5)P	1296.40	333.16	536.27	1628.56
9	21.84	865.81	2	1729.61	HexNAc(2)Hex(5)P	1296.40	434.18	637.29	1729.58
10^†^	22.41	1442.04	2	2882.07	HexNAc(2)Hex(9)P	1944.45	938.51	1141.51	2881.96
11^†^	22.74	899.33	2	1796.66	HexNAc(2)Hex(5)	1216.42	581.24	784.31	1796.66
12	23.75	881.79	2	1761.58	HexNAc(2)Hex(5)P	1296.40	466.21	669.27	1761.61
13	23.97	866.34	2	1730.68	HexNAc(2)Hex(5)	1216.42	515.24	718.29	1730.66
14^†^	24.73	971.36	2	1940.72	HexNAc(2)Hex(5)P	1296.40	645.46	848.45	1940.86
15	26.96	828.31	2	1654.62	HexNAc(2)Hex(5)P	1296.40	359.22	562.31	1654.62
16	27.08	1048.85	2	2095.71	HexNAc(2)Hex(5)P	1296.40	800.50	1003.39	2095.90
17^†^	28.38	1129.88	2	2257.76	HexNAc(2)Hex(6)P	1458.45	800.38	1003.46	2257.83
18	28.81	1052.87	2	2103.72	HexNAc(2)Hex(6)P	1458.45	646.23	849.38	2103.68
19^†^	29.04	971.85	2	1941.70	HexNAc(2)Hex(5)P	1296.40	646.21	849.40	1941.61
20	31.33	1051.40	2	2100.76	HexNAc(2)Hex(5)P	1296.40	805.57	1008.52	2100.97
21	33.85	1028.39	2	2054.81	HexNAc(2)Hex(5)P	1296.40	759.47	962.48	2054.87
22	34.74	906.32	2	1810.64	HexNAc(2)Hex(5)P	1296.40	515.17	718.29	1810.57
23	37.15	878.83	2	1755.66	HexNAc(2)Hex(5)P	1296.40	460.36	663.36	1755.76

^*^MS/MS spectra delivers information about the sugar structure *via* glycosidic bond cleavages, and provides molecular mass information for the modified peptide in the form of Y0 (peptide + H^+^) and Y1 (peptide + HexNAc(1) + H^+^) ions.

^†^Their HCD and CID spectra are shown in the Supplementary Figure (Fig. S4).

^#^Abbreviations: N-acetylhexoamine (HexNAc), Hexose (Hex), Phosphate (P).

### Both uncapping and α(1,2)-mannosidase trimming are required for proper lysosomal targeting

**Figure 2:**
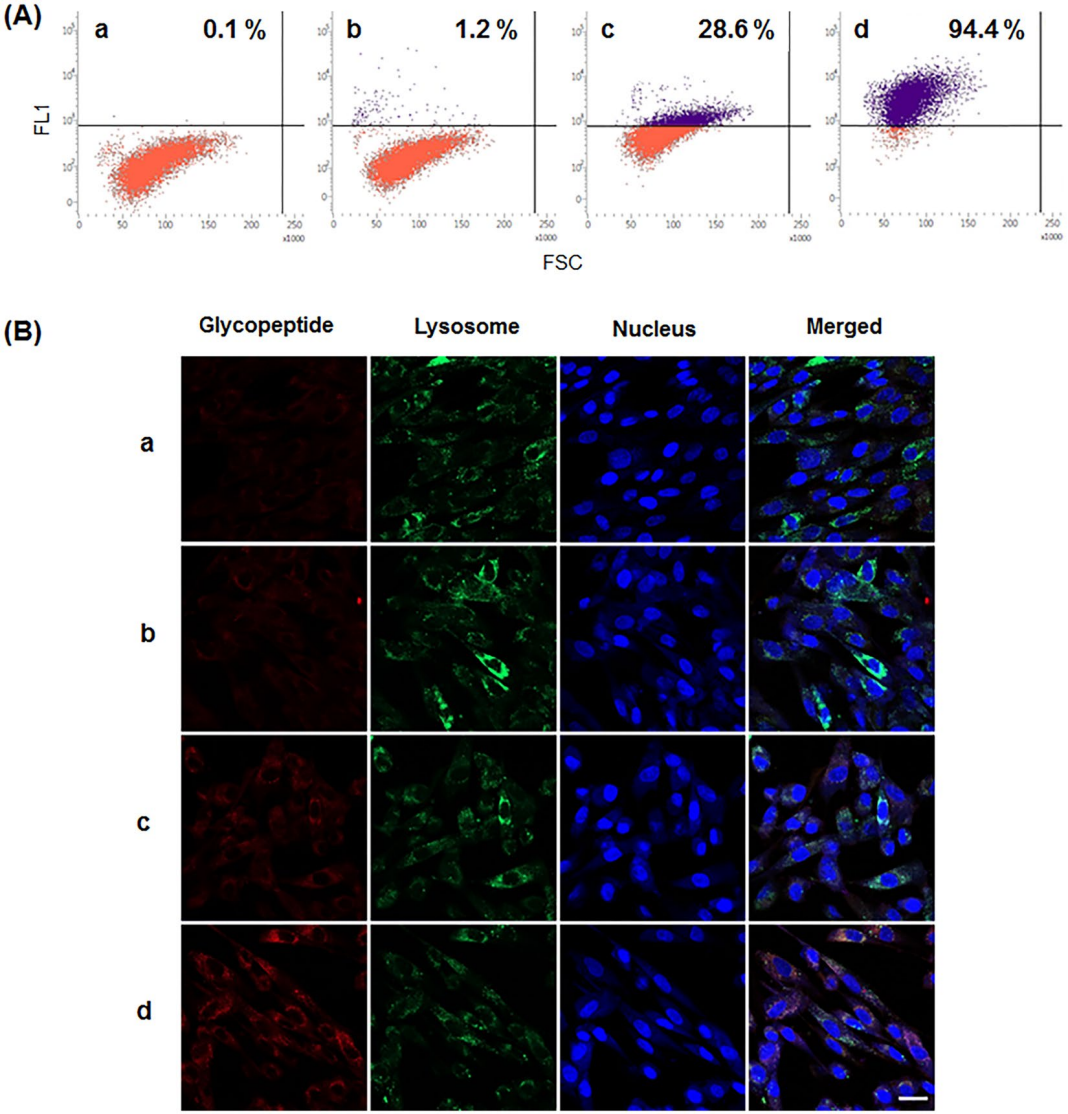
Analysis of cellular uptake and lysosome targeting capability. The glycopeptides in a – d preparation steps (Fig. 1) were labeled with biotin and then allowed to bind streptavidin-Alexa633. The resulting complexes were incubated with human fibroblasts, followed by analyses using flow cytometry (**A**) and fluorescence microscopy (**B**). In flow cytometry analysis, the population of cells displaying higher fluorescence over the threshold level is represented. In fluorescence microscopic images, the glycopeptides, lysosome, and nucleus are represented with red, green, and blue fluorescence, respectively. Scale bar = 100 µm.

For analysis of lysosomal targeting capability, the glycopeptides from the preparation steps were labeled with biotin and then bound to streptavidin-Alexa Fluor 633 conjugate (streptavidin-Alexa633). The resulting complexes of glycopeptide-biotin and streptavidin-Alexa633 were used to treat human fibroblasts for one hour, followed by a cellular uptake analysis (Fig. 2). Flow cytometry analysis shows that the glycopeptides containing phosphorylated glycans (P-Man_8_GlcNAc_2_ and P_2_-Man_8_GlcNAc_2_) without α(1,2)-mannosidase trimming as well as mannosylphosphorylated glycans were rarely taken up by human fibroblasts. A significant population (28.6%) of fibroblasts endocytosed the M6PgPs (Fig. 2A-c) only after both the outer mannose uncapping and terminal α(1,2)-mannose trimming steps. Notably, simple addition of a TiO_2_ column purification step greatly increased cellular uptake efficiency up to 94.4% (Fig. 2A-d).

Fluorescence microscopic images show that the endocytosed M6PgPs (P-Man_5–6_GlcNAc_2_ and P_2_-Man_6_GlcNAc_2_) were localized to lysosomes; the red fluorescence of M6PgPs was well merged with the green fluorescence of the lysosome marker (Fig. 2B). As shown in fluorescence microscopy analysis, only M6PgPs elaborated by both of the outer mannose uncapping and the terminal mannose trimming steps were efficiently targeted to lysosomes (Fig. 2B-c). Also, the TiO_2_ column purification step greatly increased the lysosome targeting capability (Fig. 2B-d). Taken together, pronase digestion, uncapping, mannose trimming, and TiO_2_ column purification efficiently produced M6PgPs from cell wall mannnoproteins of glyco-engineered yeast, and these M6PgPs displayed excellent lysosome targeting capability.

### Conjugation of a therapeutic enzyme with M6PgPs by using copper-free click chemistry

To prove the usefulness of the M6PgPs, we tried to attach them to a therapeutic enzyme, rGAA (alglucosidase alfa) used for treatment of Pompe disease. rGAA has a low efficacy due to a low M6P glycan content^8,11^. Popular chemical crosslinkers containing amine-reactive N-hydroxysuccinimide (NHS) esters were first employed because both rGAA and M6PgPs have primary amines in Lys residues and/or the N-terminal amino acid. However, the use of homo-bifunctional crosslinkers containing NHS ester groups at both ends was not successful and induced aggregations despite various trials with adjustments of molar ratio (data not shown).

**Figure 3:**
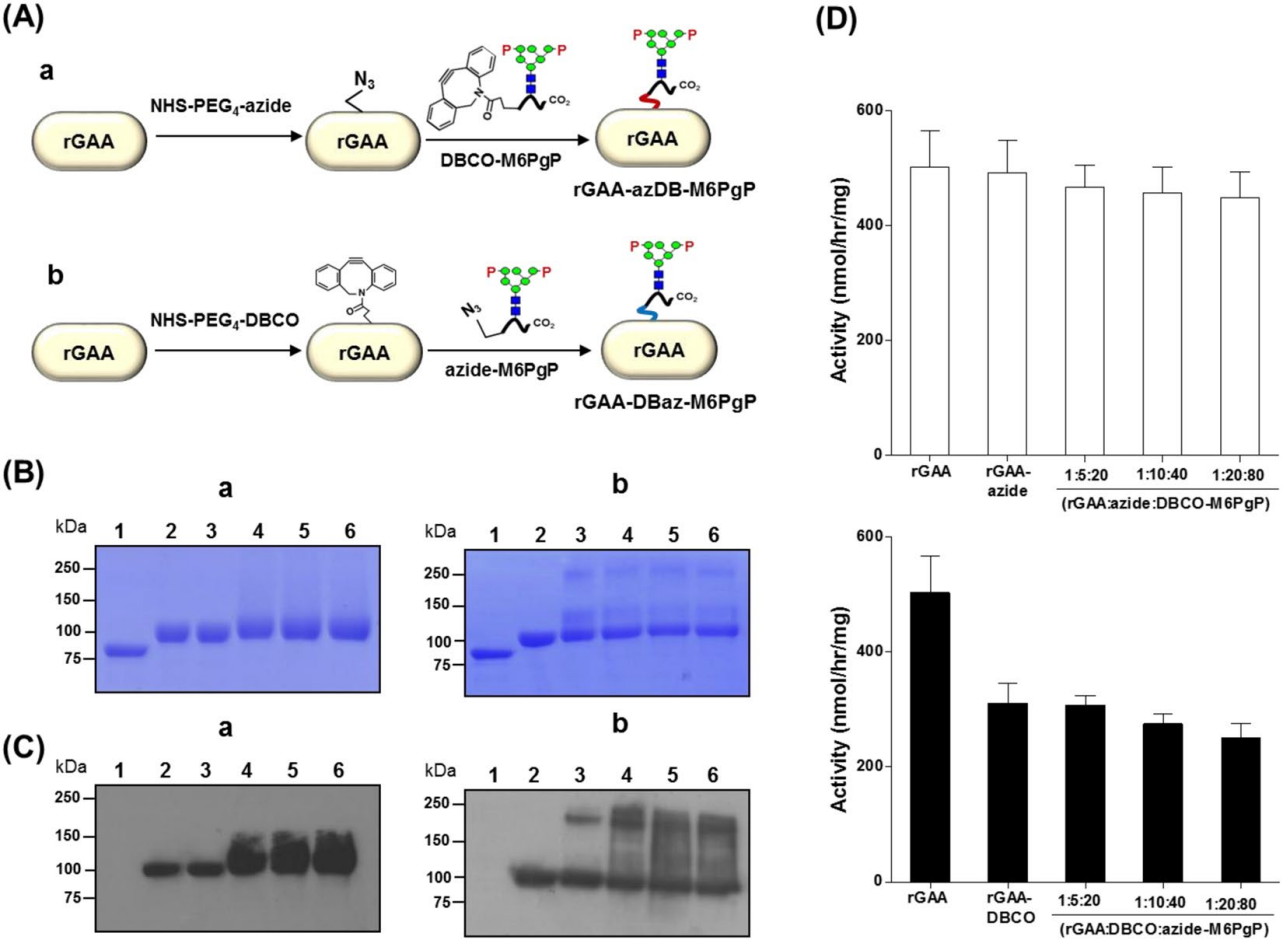
Conjugation of rGAA with M6PgPs by using copper-free click chemistry. (**A**) Two different routes to generate M6PgP-conjugated rGAA are schematically represented. In (a) route, rGAA was conjugated with NHS-PEG_4_-Azide and the resulting rGAA-azide was linked to DBCO-M6PgP made by M6PgP and DBCO-PEG_4_-NHS conjugation. This reaction product was designated as rGAA-azDB-M6PgP. In (b) route, rGAA was conjugated with DBCO-PEG_4_-NHS and the resulting rGAA-DBCO was linked to azide-M6PgP, which generated rGAA-DBaz-M6PgP. (**B**) Sizes of rGAA and its conjugates were analyzed by SDS-PAGE. (**C**) M6P glycans of rGAA and its conjugates were detected by using Dom9–3xFlag-His_8_ blotting analysis: lane 1: PNGase F-treated rGAA, lane 2; rGAA, lane 3; rGAA-azide (a) and rGAA-DBCO (b), lane 4–6; 1:5:20, 1:10:40, and 1:20:80 [rGAA:NHS-PEG_4_-Azide:DBCO-M6PgP (a) and rGAA:NHS-PEG_4_-DBCO:azide-M6PgP (b)] molar ratio reaction products. (**D**) Enzyme activities of rGAA-azDB-M6PgP and rGAA-DBaz-M6PgP are represented by white (a) and black (b) bars, respectively. The data represent the averages of three repeated experiments with standard deviations (error bars).

In order to avoid aggregation, we designed two-step reactions using two hetero-bifunctional crosslinkers (NHS-PEG_4_-Azide and NHS-PEG_4_-DBCO). First, the NHS ester of each crosslinker was reacted with primary amines of rGAA or M6PgPs to form covalent amide bonds. In the second reaction, the azide of one conjugate was linked to DBCO of the other conjugate through a copper-free click reaction, which is bioorthgonal^26^ and also well suited for protein conjugation without impairing activity and function^27^. This two-step reaction by using NHS-PEG_4_-Azide and NHS-PEG_4_-DBCO can be achieved through two different reaction routes (Fig. 3A). In the first route, after reaction of rGAA with NHS-PEG_4_-Azide, the resulting azide-functionalized rGAA (rGAA-azide) was ligated to DBCO-functionalized M6PgP (DBCO-M6PgP) prepared by conjugation of M6PgP with NHS-PEG_4_-DBCO, which generated rGAA-azDB-M6PgP (Fig. 3A-a). In the other route, rGAA-DBCO made by conjugation of rGAA with NHS-PEG_4_-DBCO was linked to azide-M6PgP, which generated rGAA-DBaz-M6PgP (Fig. 3A-b).

The M6PgP-conjugated rGAAs prepared through two different routes (rGAA-azDB-M6PgP or rGAA-DBaz-M6PgP) were analyzed by SDS-PAGE (Fig. 3B). The conjugation reactions of rGAA with crosslinkers were accomplished at three different molar ratios (1:5, 1:10, 1:20) as rGAA has 16 primary amines (15 Lys residues and one N-terminal α-amino group) at the maximum while M6PgP was conjugated with 10-fold molar excess of crosslinkers for completion of reaction. The resulting rGAA-azide or rGAA-DBCO was reacted with four fold molar excess of DBCO-M6PgP or azide-M6PgP, respectively; therefore, the used molar ratios were 1:5:20, 1:10:40, and 1:20:80 (rGAA:NHS-PEG_4_-Azide:DBCO-M6PgP or rGAA:NHS-PEG_4_-DBCO:azide-M6PgP). In the rGAA-azDB-M6PgP conjugation route, all reaction products showed one band around 100 kDa, which is a similar size as rGAA (Fig. 3B-a). In contrast, two new bands (~250 kDa and ~120 kDa), besides the ~100 kDa band, were found in rGAA-DBCO as well as rGAA-DBaz-M6PgPs (lane 3–6 of Fig. 3B-b), which suggested that the newly formed bands were generated from the first conjugation reaction of rGAA with NHS-PEG_4_-DBCO rather than the second reaction.

The M6P glycans of M6PgP-conjugated rGAAs were analyzed by blotting with domain 9 of the cation-independent MPR (Dom9), which was reported to bind M6P glycan with high affinity^17,28^. The rGAA displayed ~100 kDa band in Dom9 blots (lane 2 of Fig. 3C) while the rGAA treated with PNGase F displayed no band (lane 1 of Fig. 3C), indicating that Dom9 recognizes *N*-glycan structure. Conjugation of rGAA-azide with DBCO-M6PgP enhanced the intensities of ~100 kDa band (lane 4–6 of Fig. 3C-a). The band intensities of 1:10:40 and 1:20:80 (rGAA:NHS-PEG_4_-Azide:DBCO-M6PgP) reaction products were higher than that of the 1:5:20 reaction product. On the other hand, the rGAA-DBCO conjugation with azide-M6PgP resulted in formation of the aggregated products (lane 3–6 of Fig. 3C-b).

Since SDS-PAGE and Dom9 blotting results suggested the existence of nonspecific aggregated products, especially in rGAA-DBCO conjugates, we investigated whether rGAA conjugates can maintain GAA enzyme activity (Fig. 3D). The rGAA-azide and rGAA-azDB-M6PgPs showed similar activities with non-modified rGAA, whereas rGAA-DBCO and rGAA-DBaz-M6PgPs had significantly decreased activity. This decrease would be caused solely by conjugation of rGAA with NHS-PEG_4_-DBCO because GAA-DBCO and GAA-DBaz-M6PgP displayed similar low values. Since conjugation of rGAA with NHS-PEG_4_-DBCO induced aggregation problems involving activity decrease, the rGAA-azDB-M6PgP reaction route was selected for efficient M6PgP conjugation.

### N-Glycan analysis of M6PgP-conjugated rGAA

**Figure 4:**
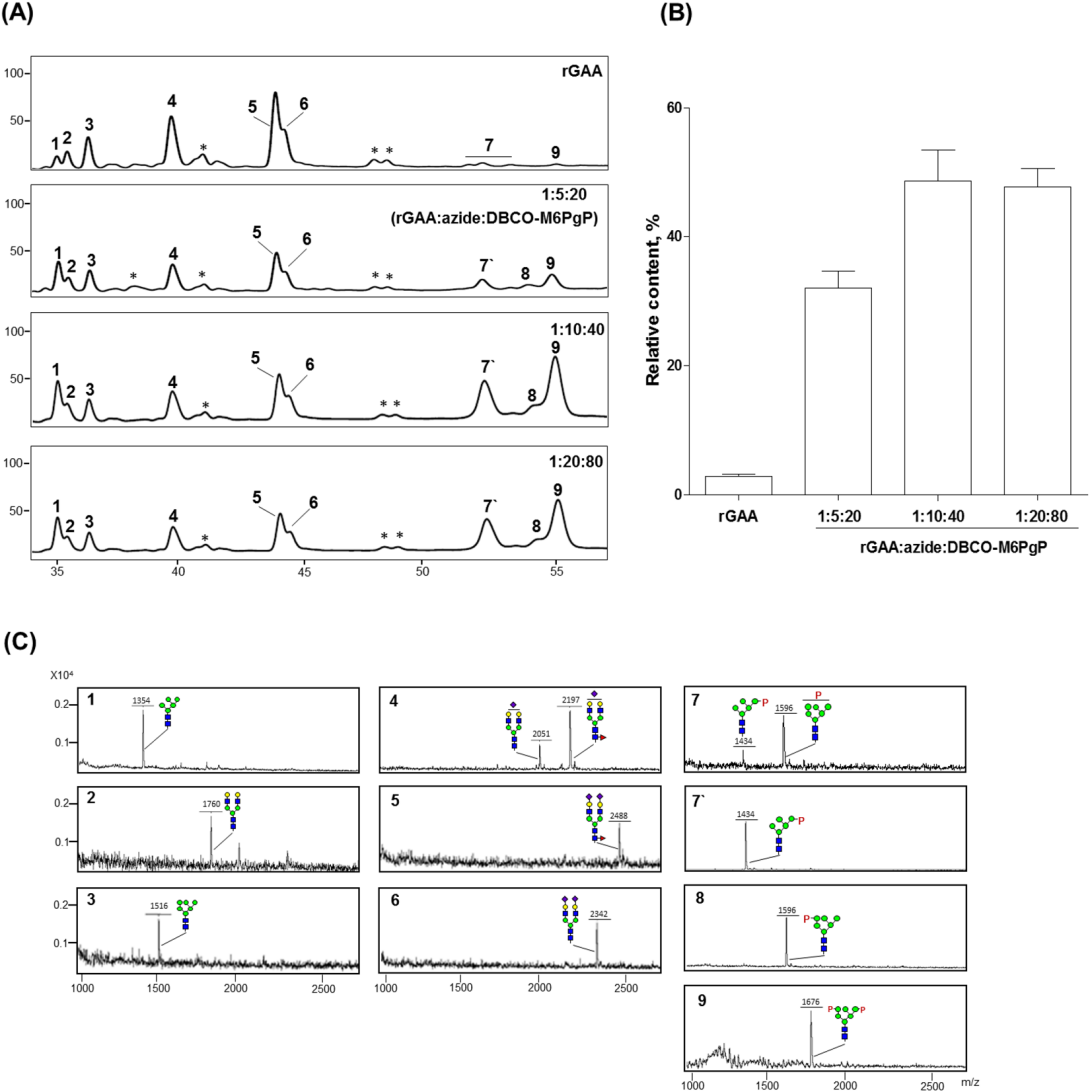
Analysis of *N*-glycans obtained from M6PgP-conjugated rGAA. *N*-Glycans of rGAA and rGAA-azDB-M6PgP (1:5:20, 1:10:40, and 1:20:80 molar ratio reactions) were analyzed by using HPLC (**A**). The masses of collected peaks were determined by using MALDI-TOF mass spectrometry (**C**). The identified glycan peaks were designated by numbers whereas unidentified peaks were represented by*. (**B**) Relative contents (%) of M6P glycan peaks (7–9) were obtained by the calculation of the integrated peak areas [100× (the area of M6P glycan peaks)/(total areas of all glycan peaks)]. The quantified data represent the averages of three repeated experiments with standard deviations. Symbols are identical to those used in Fig. 1.

*N*-glycans of rGAA and three rGAA-azDB-M6PgPs produced from the different molar ratio reactions were released by PNGase F treatment and analyzed after 2-aminobenzoic acid (2-AA) labeling by using high performance liquid chromatography (HPLC) (Fig. 4A), which was optimized for M6P glycan analysis in our previous work^19,22^. Glycan peaks separated in HPLC were collected and their masses were measured for identification by using MALDI-TOF mass spectrometry (Fig. 4C); the glycan peak identities were further confirmed by α(1,2)-mannosidase and/or alkaline phosphatase digestions (data not shown). Also, mass spectra of whole 2-AA-labeled glycans were analyzed as a crosscheck (Supplementary Fig. S5). As reported^10,12^, rGAA has high-mannose type (peak 1 and 3), non-sialylated (peak 2), and sialylated complex type bi-antennary (peak 4, 5 and 6) glycans together with very low content of M6P glycans (peak 7 and 9); relative content of the M6P glycan peaks, calculated from the integrated peak areas, was just 3 ± 0.4% (Fig. 4B). Owing to low intensities in rGAA glycan analysis, the mono-phosphorylated glycan peaks in close location were collected together and assigned as the peak 7 (P-Man_5_GlcNAc_2_ and P-Man_6_GlcNAc_2_), while the peak 9 was assigned as bi-phosphorylated glycan (P_2_-Man_6_GlcNAc_2_). The *N*-glycan profile obtained from M6PgP-conjugated rGAA showed that the intensities of M6P glycan peaks greatly increased. Here, the masses of peak 7′ and 8 correspond to P-Man_5_GlcNAc_2_ and P-Man_6_GlcNAc_2_ glycans, respectively. Because peak 8, observed only in M6PgP-conjugated rGAA, was generated by α(1,2)-mannosidase digestion, its phosphorylation site should be located at the terminal mannose of α(1,3)-branch. The highest relative content (49 ± 5.1%) of M6P glycans was observed in M6PgP-conjugated rGAA generated by the 1:10:40 (rGAA:NHS-PEG_4_-Azide:DBCO-M6PgP) molar ratio reaction while the 1:5:20 and 1:20:80 reaction products displayed 32 ± 4.1% and 48 ± 4.3% M6P glycan contents, respectively. Therefore, in the following experiments, we used M6PgP-conjugated rGAA generated by the 1:10:40 reaction.

### M6PgP-conjugated rGAA uptake by fibroblasts of Pompe disease patient

**Figure 5:**
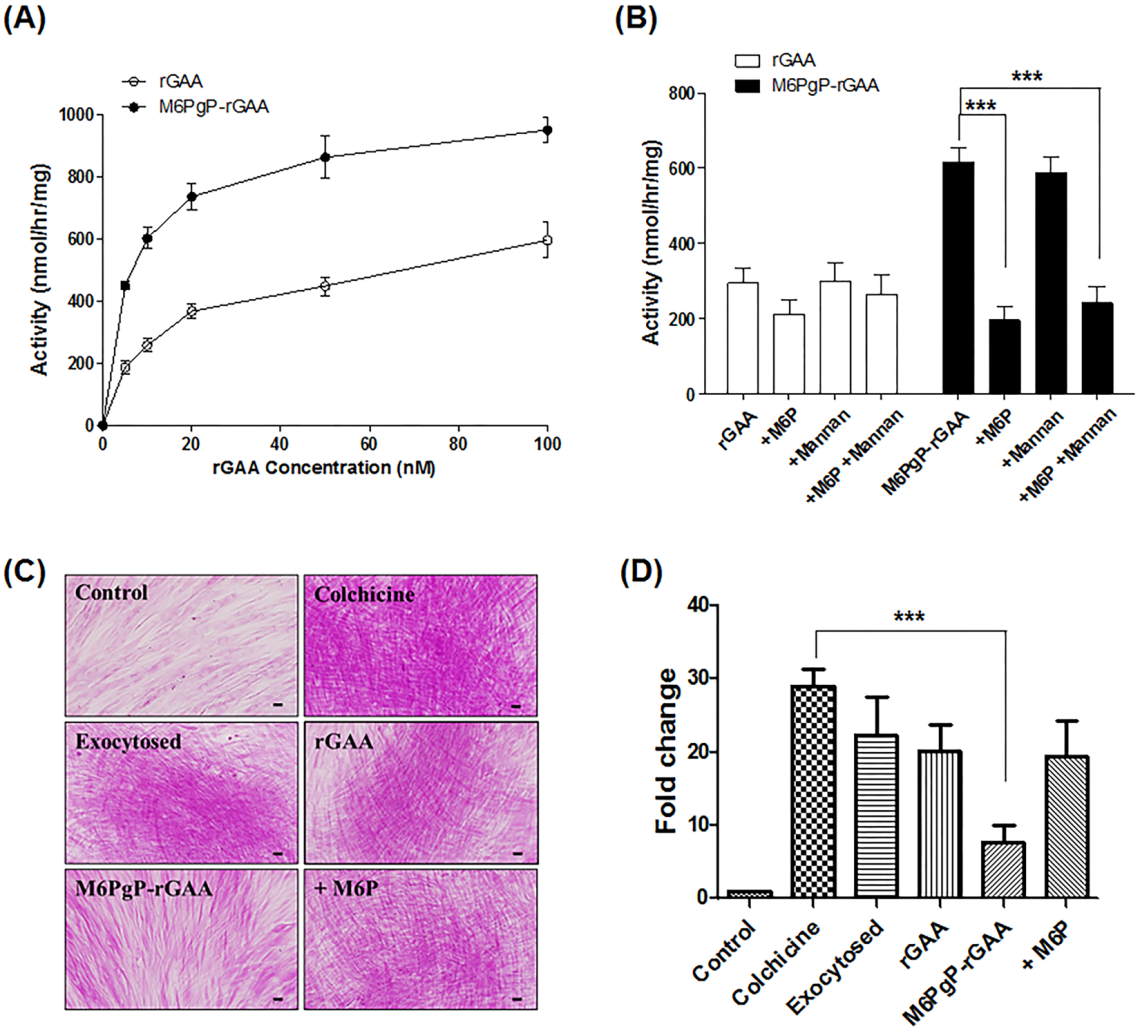
Enhanced uptake of M6PgP-conjugated rGAA by Pompe disease patient fibroblasts. (**A**) Intracellular GAA activities of Pompe disease patient fibroblasts were analyzed after incubation with increasing concentrations of rGAA (open circle) and M6PgP-conjugated rGAA (M6PgP-rGAA; closed circle). (**B**) The fibroblasts were incubated with 20 nM of rGAA (white bar) or M6PgP-rGAA (black bar) in the presence or absence of 5 mM M6P and/or 2 mg/ml mannan. Data are shown as mean ± standard deviation (n = 3). (**C**) Representative images of PAS-stained Pompe fibroblasts (magnification: x 400). Glycogens were accumulated in the cells cultured in the presence of colchicine (Colchicine). The accumulated glycogens slightly decreased through exocytosis in the absence of colchicine (Exocytosed) and were further digested by addition of 100 nM rGAA (rGAA) or M6PgP-conjugated rGAA (M6PgP-rGAA). Free M6Ps competitively inhibited glycogen clearance by M6PgP-conjugated rGAA (+M6P). Scale bar = 50 µm. (**D**) The images of PAS-stained cells in each well of 12-well culture plates were analyzed by using NIH Image J software (http://rsb.info.nih.gov/ij/). Strong purple spots over the arbitrary threshold value were identified and their areas were used for calculation of relative fold changes in comparison with the strong purple spot area of control cells (Supplementary Fig. S6). Data are shown as mean ± standard deviation (n = 4). One-way ANOVA with Tukey’s multiple comparison test, ^***^P < 0.001.

The uptakes of M6PgP-conjugated rGAA and unmodified rGAA by Pompe disease patient fibroblasts were compared by measuring intracellar GAA activity after incubation with increasing concentrations of the enzyme (Fig. 5A). M6PgP-conjugated rGAA showed much higher activities at all of the measured concentrations. Moreover, the highest activity displayed by 100 nM of rGAA was similar to that by 10 nM of M6PgP-conjugated rGAA. The intracellular GAA activities enhanced by 20 nM M6PgP-conjugated rGAA treatment were greatly reduced by addition of free M6P (5 mM) and not by mannan (2 mg/ml) (Fig. 5B), indicating that the uptake was achieved by MPR-mediated endocytosis.

The deficiency of lysosomal enzyme GAA in Pompe disease patient led to glycogen accumulation in many tissues including skeletal muscle and heart. However, most of the stored glycogen is exocytosed from the cultured fibroblasts of Pompe patient^29^. When the Pompe fibroblasts were examined by using periodic acid-schiff (PAS) staining, cell surfaces were stained and only a few purple spots were observed inside cells (Fig. 5C). However, addition of colchicine, which was reported to inhibit glycogen exocytosis^30^, greatly increased the number and intensity of strong purple spots in Pompe fibroblasts (Fig. 5C), which indicates that exocytosis inhibition induced glycogen accumulation. The areas of strong purples spots were quantitatively analyzed from the images of the PAS-stained cells in 12-well plates (Supplementary Fig. S6), followed by calculation of relative fold change in comparison with the strong purple spot area of control cells (Fig. 5D). The cells cultured in the presence of colchicine for five days showed the highest fold change of area (29.0 ± 2.3), which indicated the accumulation of glycogen. Further culture in the medium without colchicine for two days resulted in the slightly decreased fold change value (22.3 ± 5.3), which would be caused by spontaneous exocytosis of glycogen. When the cells were further cultured in the medium containing 100 nM rGAA, the fold change value of strong purple spot area was 20.2 ± 3.6, which is a statistically insignificant decrease. In contrast, the further culture in the medium containing 100 nM M6PgP-conjugated rGAA showed the significantly decreased value (7.7 ± 2.3), clearly indicating that M6PgP-conjugated rGAA was much more efficient in removing the accumulated glycogen in the Pompe fibroblasts. The value dropped by M6PgP-conjugated rGAA treatment was reversed up to 19.5 ± 4.8 by addition of 5 mM free M6P, indicating that the clearance of accumulated glycogen is achieved by MPR-mediated endocytosis of M6PgP-conjugated rGAA.

## Discussion

Chemical conjugation of synthetic M6P glycan was successful in increasing M6P glycan content of rGAA, which enhanced lysosomal targeting and therapeutic efficacy^5,9–12^. However, complex and laborious multistep procedures for M6P glycan synthesis were required including repetitive protection, deprotection, and purification steps^12,14,15^. Instead of synthetic M6P glycans, we used M6PgPs prepared from cell wall mannoproteins of glyco-engineered yeast, which has a great advantage in not requiring laborious M6P glycan synthesis procedures. Large quantities of cell wall mannoproteins, a major component of yeast cell wall, can be easily extracted from the cultured yeasts. Short glycopeptides generated from the cell wall mannoproteins by pronase digestion contain very high content of mannosylphosphorylated glycans, which were converted to M6P glycans by outer mannose uncapping. Tiels *et al*. produced the rGAA containing a high content of M6P glycans from glyco-engineered *Y. lipolytica*^7^, which is more simple and direct approach if its expression level is high enough for commercial production. We tried to produce therapeutic enzymes from glyco-engineered *S. cerevisiae*, but their secretory expression levels were disappointedly low (data not shown). M6PgP conjugation strategy can detour the problem of low secretory expression level because M6PgPs can be easily prepared from large amount of yeast cell wall proteins. It has another advantage that M6PgP can be conjugated to enzymes expressed from any hosts including mammalian cells, insect cells, plant cells, and even *Escherichia coli*.

We analyzed cellular uptake and lysosomal targeting capabilities of the glycopeptides in the preparation steps (Fig. 2). The results clearly showed that, as well as the outer mannose uncapping step, the α(1,2)-mannosidase trimming step is also an essential requirement for proper lysosome targeting, which is in accord with a previous report^7^. After the uncapping step, the glycopeptides harbored the glycans containing 8 mannose residues (Man_8_GlcNAc_2_, P-Man_8_GlcNAc_2_, and P_2_-Man_8_GlcNAc_2_); while the α(1,2)-mannosidase digestion removed terminal α(1,2)-mannose residues, which generated Man_5_GlcNAc_2_, P-Man_5–6_GlcNAc_2_, and P_2_-Man_6_GlcNAc_2_ glycans. As the removed mannose residues were located outside of the phosphorylation site, α(1,2)-mannosidase digestion would expose M6P moiety to enable the interaction with MPR on the plasma membrane of cells. After the uncapping and terminal mannose trimming steps, we added TiO_2_ chromatography for selective purification of M6PgPs, which greatly increased the efficiency of cellular uptake and lysosomal targeting capabilities.

In this study, we used the M6PgPs generated by pronase digestion because they have an N-terminal α-amino group which can be employed by many commercially available crosslinkers. Although pronase can hydrolyze all peptide bonds, pronase digestion has been known to generate short glycopeptides containing 2–6 amino acids because of the steric hindrance caused by bulky glycans^21^. We performed the proteomics experiment using LC-MS/MS spectrometry to analyze the M6PgPs derived from cell wall mannoproteins of glyco-engineered *S. cerevisiae* strain. In contrast to the previous work using a single model glycoprotein^21^, the exact amino acid sequences of the glycopeptides could not be identified from our experiment because pronase digestion generated very short peptides without specific cleavage sites. Instead, we could estimate the approximate length of peptides by dividing the obtained peptide masses with the average molecular weight of amino acids (110 Da). This simple calculation suggests that peptide lengths of M6PgPs ranged from ~2.5 to ~8.5 amino acids, which is slightly larger than the values reported in the previous work^21^. Considering that antigenic T-cell epitopes are longer than 8–10 amino acids^31,32^, the possibility that the M6PgPs elicit an immune reaction through T-cell based immunity appeared low. However, because linear B-cell epitopes have been known to have various peptide lengths from 2 to 829 residues^33^, the M6PgPs can be B-cell epitopes, which might result in antibody-driven immunity and can be a possible limitation of M6PgP-conjugation technology for enzyme replacement therapy. The efficacy and safety of M6PgP-conjugated rGAA including the potential to elicit the immune response need to be confirmed by further *in vivo* experiments.

The quality control issue caused by the heterogeneity of M6PgPs would be a challenge when pursuing clinical development. The proteomics experiment using LC-MS/MS spectrometry showed the existence of 23 glycopeptides in M6PgPs, which will make the characterization of the end product (M6PgP-conjugated rGAA) difficult. We also found the minor amounts of six glycopeptides containing *O*-linked mannoses or phosphomannoses, which increased heterogeneity. Besides, if a negatively charged component containing amine group exists in the yeast cell wall, it would be a contaminant which can be conjugated to rGAA. Due to the heterogeneity problem, the quality control system to achieve batch-to-batch consistency of M6PgP-conjugated rGAA production should be developed for the approval by the regulatory agencies.

We measured the glycopeptide concentrations by both of fluorescamine assay and monosaccharide composition analysis. Fluorescamine reacts with primary amine to generate fluorescence while the amount of GlcNAc was measured in monosaccharide composition analysis after acid hydrolysis; all of the glycans analyzed in this study have two GlcNAc residues and varying numbers of mannose residues. The concentrations of M6PgP stock solution measured by these two methods were similar (less than 10% differences); fluorescamine assay and monosaccharide composition analysis generated 1.06 mM and 0.95 mM, respectively.

M6PgPs were successfully attached to a therapeutic enzyme, rGAA, by using two commercially available crosslinkers, NHS-PEG_4_-Azide and NHS-PEG_4_-DBCO. Many other crosslinkers tested in this study induced inactivation of rGAA enzyme activity. Notably, only NHS-PEG_4_-Azide enabled rGAA to maintain the solubility and activity after crosslinking, whereas NHS-PEG_4_-DBCO failed to do this. It seems that inappropriate conjugation of Lys residues mainly located on the surface of rGAA can destabilize the protein tertiary structure, which was also previously reported by Genzyme researchers; conjugation of aromatic aldehyde-modified Lys with hydrazine-modified synthetic M6P glycan decreased the stability of the resulting rGAA conjugates^12^. Upon conjugation to rGAA, NHS-PEG_4_-DBCO, which contains an aromatic ring, induced aggregation and decreased enzyme activity; whereas NHS-PEG_4_-Azide, which has no hydrophobic moiety, did not show any harmful effect. Taken together, attachment of hydrophobic group to Lys residues of rGAA appeared to destabilize the protein tertiary structure and decrease enzyme activity, suggesting intolerance against hydrophobicity increase^12^. On the other hand, M6PgP conjugation with NHS-PEG_4_-DBCO did not cause any problem. It seems that strong hydrophilicity of M6PgP would compensate for the hydrophobicity of the DBCO group. Therefore, in our test for two different conjugation routes, only rGAA-azDB-M6PgP was successful for generating M6PgP-conjugated rGAA without an aggregation problem and loss of enzyme activity.

Because rGAA has 15 Lys residues and one N-terminal amino group, we expected that M6PgP-conjugated rGAA generated by 1:20:80 (rGAA:NHS-PEG_4_-Azide:DBCO-M6PgP) molar ratio reaction would have the highest content of M6P glycans. However, the 1:10:40 reaction product showed the maximum value in the *N*-glycan analysis result although the difference between the 1:10:40 and 1:20:80 reaction products was small. It is speculated that all of the available primary amines, located on the surface of rGAA, are conjugated in the 1:10:40 reaction because some of the Lys residues may fold into the interior of the protein.

M6PgP conjugation increased M6P glycan content up to ~49%, which is an enormous increase compared to a small amount (~3%) of unmodified rGAA. On the other hand, the intracellular activity of M6PgP-conjugated rGAA incorporated into the Pompe patient fibroblast increased only ~1.6-fold higher than the one of unmodified rGAA. However, 10 nM of M6PgP-conjugated rGAA displayed similar intracellular GAA activity as 100 nM of unmodified rGAA, suggesting that M6PgP-conjugated rGAA can be used at a ~10-fold lower dose compared to rGAA. It is similar to the previous report^7^; yrhGAA, which was produced from the glyco-engineered *Y. lipolytica* to harbor >80% M6P glycans, showed only ~2-fold increase of intracellular GAA activity but the enzyme concentration required to reach half-maximal saturation of intracellular enzyme activity was ~18-fold lower than rGAA.

During the *N*-glycan analysis of rGAA and M6PgP-conjugated rGAA, we found that M6PgP conjugation significantly increased the Man_5_GlcNAc_2_ glycan peak (peak 1 in Fig. 4A). Its relative content was 4.7 ± 1.1% in rGAA while increased up to 9.9 ± 3.0%, 10.4 ± 2.1%, and 13.3 ± 2.5% in 1:5:20 (rGAA:NHS-PEG_4_-Azide:DBCO-M6PgP), 1:10:40, and 1:20:80 reaction products, respectively. It appeared that some of the phosphate groups were removed from M6PgPs during the conjugation reactions because the Man_5_GlcNAc_2_ glycan peak was found after the DBCO conjugation of M6PgP whereas it was not in the TiO_2_*-*purified M6PgP (Supplementary Fig. S7). The TiO_2_ chromatography seemed to work well because we did not found major peaks of high-mannose type glycans without phosphate group in both MALDI-TOF mass spectrometry and HPLC analyses. However, it is also notable that several glycopeptides containing non-phosphorylated glycans were identified in LC-MS/MS proteomics analysis (Table 1 and Supplementary Table S1). It might be possible that LC-MS/MS analysis is more sensitive to detect minor glycopeptides or that some of the phosphate groups were lost during the analysis condition.

Although Pompe patients have accumulated glycogens in many tissues, PAS staining showed that the fibroblasts derived from a Pompe patient rarely accumulated glycogen (Fig. 5C). This seems to be related with the report that glycogen is exocytosed from the fibroblasts^29^. Colchicine, a cytoskeletal destabilizer inhibiting exocytosis, was shown to accumulate glycogen in Pompe fibroblasts^30^. We succeeded in PAS-staining the accumulated glycogen in Pompe fibroblasts cultured in the presence of colchicine. Although the unmodified rGAA treatment lowered the PAS-staining, the decreased glycogen level was not so significant compared with the cells experiencing spontaneous exocytosis by removal of colchicine. The accumulated glycogen was significantly removed only by M6PgP-conjugated rGAA treatment. It was also confirmed that the glycogen clearance enhanced by M6PgP conjugation was induced by MPR-mediated cellular uptake and lysosomal targeting through free M6P competition analysis.

In summary, we used the cell wall of the glyco-engineered yeast as a natural M6P glycan source for conjugation to a therapeutic enzyme because this has the great advantage of obviating the need for repetitive and laborious steps for chemical synthesis of M6P glycans. The M6PgPs were successfully conjugated to a therapeutic enzyme without enzyme activity loss through a two-step reaction using amine coupling and copper-free click chemistry. The M6PgP-conjugated rGAA showed greatly increased cellular uptake and efficient clearance of the accumulated glycogen, which suggests that our approach would lead to easy development of next-generation therapeutic enzymes with enhanced lysosomal targeting and therapeutic efficacy.

## Materials and Methods

### Preparation of M6PgPs from glyco-engineered yeast

M6PgPs were prepared from the cell wall mannoproteins of the *S. cerevisiae och1Δmnn1Δ* strain transformed with the YEP352-YlMpo1 plasmid^18^. First, the ~20 mg of yeast cell wall mannoproteins (wet cell weight) were extracted with hot citrate buffer, as described previously^18^. The obtained mannoproteins (~1 mg) were digested with 0.1 mg of pronase at 55 °C for 48 h. From the 1 ml of resulting reaction mixture, short glycopeptides were purified by using solid-phase extraction using graphitized carbon columns (Alltech, Lexington, MA) as previously described^34^. The mannosylphosphorylated glycans of glycopeptides were uncapped by MAH; 0.5 M formic acid at 80 °C for 1 h^19,22^. After drying in a speedvac, the uncapped glycopeptides (~0.2 mg) were treated with 10 mU of α(1,2)-mannosidase in 20 mM ammnonium acetate (pH 5.0) at 37 °C for 16 h. Finally, the M6PgPs were selectively purified by using Titanosphere (TiO2) beads (5 µm chromatographic material; GL Sciences, Japan), as previously described^24^ with a slight modification. Briefly, the whole glycopeptide fraction was loaded on TiO_2_ beads in loading buffer (1 M glycolic acid in 80% ACN, 5% TFA), followed by incubation at room temperature (RT) for 30 min. After the removal of supernatant, the beads were thoroughly washed by using washing buffer 1 (80% ACN, 1% TFA) and 2 (80% ACN, 0.1% TFA). The M6PgPs were eluted from the TiO_2_ beads by using elution buffer (28% ammonium hydroxide solution). The concentrations of the glycopeptides were measured by using a fluorescamine fluorescence assay^35^ and monosaccharide composition analysis^36^. For the fluorescamine assay, the glycopeptides eluted from the TiO_2_ beads were dried in a speedvac and then were redissolved in 160 μl of 0.5 M sodium borate buffer (pH 8.0). While the solutions were vortexed vigorously, 40 μl of 0.3 mg/ml fluorescamine (Sigma-Aldrich, St. Louis, MO) in acetone was rapidly added. The fluorescence was analyzed on a SpectraMax i3 plate reader (Molecular Devices, San Jose, CA) with 390 nm excitation and 470 nm emission (10 nm bandwidth). For the monosaccharide composition analysis, the carbohydrates released from glycopeptides by acid hydrolysis were labeled with 2-AA, and then analyzed by using reverse phase HPLC with fluorometric detection as described previously^36^. Monosaccharide standard solutions (Prozyme, Hayward, CA) were used for peak identification and quantification.

### LC-MS/MS for glycopeptide analysis

The purified glycopeptide sample was dissolved in 20 μl of water containing 0.1% formic acid (Sigma-Aldrich) and analyzed using an LC-MS/MS system consisting of an EASY-nanoLC 1100 System (ThermoFisher Scientific, Waltham, MA) and an LTQ Orbitrap Elite mass spectrometer (ThermoFisher Scientific) equipped with an EASY-Spray source. Briefly, six µl aliquots of the peptide solutions were loaded into Acclaim PepMap 100 (75 µm × 2 cm, nanoViper). Peptides were desalted for 4 min at a flow rate of 4 μl/min. Then, the trapped peptides were separated on a PepMap RSLC column (C_18_ particle size 2 μm, 100 A, 75 μm × 50 cm). The mobile phases, A and B, were composed of 0 and 100% acetonitrile, respectively, and each contained 0.1% formic acid. The LC gradient began with 5% B for 1 min and was ramped to 8% B over 16 min, to 40% B over 74 min, and to 95% B over 1 min. Then, it remained at 95% B over 8 min and was decreased to 5% B for another 5 min. The column was re-equilibrated with 2% B for 15 min before the next run. The voltage applied to produce an electrospray was 2.2 kV. During the chromatographic separation, the LTQ Orbitrap Elite was operated in a data-dependent mode. The MS data were acquired using the following parameters: five data-dependent collision induced dissociation-high energy collision dissociation (CID-HCD) dual MS/MS scans per full scan; CID scans were acquired in linear trap quadrupole (LTQ) with two-microscan averaging; full and HCD scans were acquired in Orbitrap at resolution 120,000 and 15,000, respectively, with two-microscan averaging; 35% normalized collision energy (NCE) in CID and 35% NCE in HCD; ± 5 Da isolation window. Previously fragmented ions were excluded for 30 sec. In CID-HCD dual scan, each selected parent ion was first fragmented by CID and then by HCD.

All MS and MS/MS spectra were manually identified as follows. First, the glycopeptides were selected from HCD and CID MS/MS spectra based on the presence of unique oxonium ions from glycan fragmentation. There are ions of hexose with phosphate [m/z 243.03 of Hex(1)Pi(1), 405.03 of Hex(2)Pi(1) and 770.22 of HexNAc(1)Hex(3)Pi(1)] and without phosphate [(m/z 325.11 of Hex(2) and 690.24 of HexNAc(1)Hex(3)]. Second, we confirmed the presence of B-type fragment ions by the neutral loss of the peptide with HexNAc(1) from the glycopeptide precursor ions in CID MS/MS spectra. Finally, in order to determine the molecular weight of peptide, at least two Y-type ions were confirmed^37^.

### Biotin labeling of glycopeptides and cellular uptake analyses

The glycopeptides in each of the M6PgP preparation steps were biotinylated with the Biotin Protein Labeling Kit (Roche, Basel, Switzerland); 50 μM of glycopeptides were incubated in phosphate-buffered saline (PBS) with 10-fold molar excess of biotin-7-NHS for 2 h at room temperature (RT) and non-reacted labeling reagents were removed by dialysis over an one kDa cutoff membrane. The 2.5 μM of biotinylated glycopeptides were allowed to bind 10 μg/ml of streptavidin-Alexa633 (ThermoFisher Scientific) in 0.2 ml Dulbecco’s Modified Eagle Medium (DMEM) for 1 h at RT. The resulting solutions were incubated with human fibroblasts which were cultured in DMEM supplemented with 10% fetal bovine serum (FBS), at 3 × 10^4^ cells/well in μ-slide 8-well plates (Ibidi, Verona, WI) in a 5% CO_2_ incubator at 37 °C. After 1 h incubation of the glycopeptide-streptavidin complex with human fibroblasts, the cells were extensively washed with PBS and then analyzed on a FACSVerse flow cytometer (BD Biosciences, San Diego, CA).

For fluorescent microscopic image analysis, after 1 h incubation with the glycopeptide-streptavidin complex (2.5 μM biotinylated glycopeptides and 10 μg/ml streptavidin- Alexa633), the fibroblasts were washed with PBS and then stained with 50 nM LysoTracker green (ThermoFisher Scientific) for 1 h at 37 °C. After PBS washing, the cells were fixed in 2% paraformaldehyde for 20 min at RT, followed by nuclei staining using 4′-6 diamidino-2-phenylindole (DAPI) (ThermoFisher Scientific). Fluorescence images of the stained cells were analyzed using a Zeiss LSM510 confocal microscope (Carl Zeiss, Oberkochen, Germany).

### Conjugation of rGAA with M6PgPs by using two hetero-bifunctional crosslinkers

The rGAA, aglucosidase alfa (Myozyme, Sanofi Genzyme, Boston, MA, USA), was conjugated with NHS-PEG_4_-Azide (Pierce, Rockford, IL, USA) or NHS-PEG_4_-dibenzocyclooctyne (DBCO) (Click Chemistry Tools, San Diego, CA). Crosslinker stock solutions (200 mM) prepared in dimethyl sulfoxide (DMSO) were diluted to 200 µM of rGAA solution in 100 µL PBS for the conjugation reaction with 5-fold, 10-fold, or 20-fold molar excess. After 3 h incubation at RT, non-reacted crosslinkers were removed by dialysis in PBS over a 30 kDa cutoff membrane. Likewise, the M6PgPs were also conjugated with NHS-PEG_4_-DBCO or NHS-PEG_4_-Azide. Crosslinker stock solutions (200 mM) prepared in DMSO were diluted to 1 mM of M6PgP solution in 200 µL PBS for the conjugation reaction with 10-fold molar excess. After 3 h incubation at RT, non-reacted crosslinkers were removed by dialysis in PBS over a 1 kDa cutoff membrane (Spectrum Labs). The resulting M6PgP conjugates were dried in a speedvac and made to 50 mM stock solution by re-dissolving it in PBS. In the second reaction, azide-conjugated and DBCO-conjugated rGAA were incubated, respectively, with four-fold molar excess of DBCO-conjugated and azide-conjugated M6PgPs in PBS for 4 h at 37 °C, which generated 1:5:20 (50 µM rGAA: 250 µM NHS-PEG_4_-Azide: 1 mM DBCO-M6PgP and 50 µM rGAA: 250 µM NHS-PEG_4_-DBCO: 1 mM azide-M6PgP), 1:10:40 (50 µM rGAA: 500 µM NHS-PEG_4_-Azide: 2 mM DBCO-M6PgP and 50 µM rGAA: 500 µM NHS-PEG_4_-DBCO: 2 mM azide-M6PgP), and 1:20:80 (50 µM rGAA: 1 mM NHS-PEG_4_-Azide: 4 mM DBCO-M6PgP and 50 µM rGAA: 1 mM NHS-PEG_4_-DBCO: 4 mM azide-M6PgP) conjugation products. Unreacted azide- or DBCO-conjugated M6PgPs were removed by dialysis in PBS using a 30 kDa cutoff membrane.

### *In vitro* GAA activity assay

The GAA enzyme activity was assayed by using the substrate 4-methylumbelliferyl-α-D-glucopyranoside (4-MUG) (Sigma-Aldrich) which generates fluorescence on digestion. The 50 µl of the reaction mixture (0.2 M sodium acetate, 0.4 M potassium chloride, pH 4.3) including one µg of rGAA or its conjugates were incubated for 30 min at 37 °C. The reaction was terminated by adding 150 µl of 100 mM glycine/NaOH buffer (pH 11) and the fluorescence was measured by using a plate reader SpectraMax i3 (Molecular Devices) with 375 nm excitation and 460 nm emission.

### Dom9 blotting for detection of M6P glycans

The gene coding domain 9 (Dom9) of the cation-independent M6P receptor was synthesized with codon optimization for recombinant expression in *Pichia pastoris* and cloned to the pD912 vector by the gene synthesis and cloning service of ATUM (Newark, CA). Three tandem Flag-tags and a His_8_-tag were added to the C-terminus of Dom9 (Dom9–3xFlag-His_8_); the whole amino acid sequences including the α-mating factor for secretion are represented in Supplementary Fig. S8. The vector pD912-Dom9 was transformed to *P. pastoris* GS115 strain and the transformants were selected on YPD (1% yeast extract, 2% Bacto peptone, 2% glucose) containing 0.1 mg/ml Zeocin. A single colony was initially cultured in YPD medium containing 0.1 mg/ml Zeocin, which was transferred to flasks containing BMMY (1% yeast extract, 2% peptone, 100 mM potassium phosphate, pH 6.0, 1.34% YNB, 0.00004% biotin, 0.5% MeOH) medium for methanol induction and further cultured for 3 days at 30 °C; Dom9 overexpression was maintained by adding 0.5% methanol every 24 h. Dom9–3xFlag-His_8_ protein was purified from the culture supernatant by using Ni-NTA column chromatography as described previously^34,38^.

After standard sodium dodecyl sulfate-polyacrylamide gel electrophoresis (SDS-PAGE) using 8% polyacrylamide gel, rGAA and its conjugates were transferred to a PVDF membrane. After a blocking step using 5% skim milk in Tris-buffered saline (50 mM Tris-Cl, pH 7.5, 150 mM NaCl) containing 0.1% Tween-20 (TBST), the membrane was incubated with 0.5 μg/ml of Dom9–3xFlag-His_8_ in TBST for 2 h. After three times repeated washing steps, the membrane was incubated with a horseradish peroxidase-conjugated anti-Flag antibody (Sigma-Aldrich) in TBST for 1 h. After extensive washing steps, the resulting membrane was visualized by using ECL Western blot detection kit (GE Healthcare Life Sciences, Chicago, IL).

### N-Glycan analysis

*N*-Glycans were released from the glycopeptides or rGAA by PNGase F (New England Biolabs, Ipswich, MA) treatment and purified by solid-phase extraction using graphitized carbon columns as described previously^34,39^. After labeling with 2-AA, the labeled glycans were purified from unreacted reagents using cyano-SPE cartridge (Agilent Technologies, Santa Clara, CA) as previously described^19,38^. The 2-AA-labeled glycans were analyzed on a Shodex Asahipak NH2P-50 4E (4.6 × 250 mm) column (Showa Denko, Tokyo, Japan) using a Waters Alliance system equipped with a Waters 2475 fluorescence detector (Milford, MA) in the conditions optimized for M6P glycan analysis as previously described^19,22^. The eluted fractions corresponding to the glycan peaks were collected, and their masses were identified by MALDI-TOF mass spectrometry (Microflex, Bruker Daltonik, Bremen, Germany), as described previously^18,22^.

### Cellular uptake and intracellular GAA activity assay

The fibroblasts cells (GM00248) of Pompe patient (Pompe fibroblast), who had homozygous mutation in exon 18 of the GAA gene leading to formation of premature stop codon at 854, were obtained from the Coriell Institute (https://catalog.coriell.org/). Pompe fibroblasts were seeded at 3 × 10^4^ cells/well in 24 well culture dishes in DMEM supplemented with 10% FBS and allowed to settle for 24 h at 37 °C. Various concentrations (0–100 nM) of rGAA and its conjugates were added to the cells and incubated for 18 h at 37 °C; for inhibition assay, 5 mM of M6P (GeneChem, Daejeon, Korea) and 2 mg/ml of mannan (Sigma-Aldrich) were added. After incubation, the cells were washed first with PBS containing 1 mM M6P and then with PBS alone. The cells were harvested with 0.25% trypsin and resuspended in GAA assay buffer (0.2 M sodium acetate, 0.4 M potassium chloride, 0.1% triton X-100, pH 4.3). For cell lysis, cell suspensions were frozen at −80 °C for 30 min, followed by thawing at 37 °C. The resulting cell lysates were centrifuged at 14,000 g for 10 min to remove cell debris. The supernatants were assayed by using 4-MUG as described in ‘*in vitro* GAA activity assay’ subsection.

### Glycogen exocytosis inhibition and periodic acid-schiff (PAS) staining

Pompe fibroblasts were seeded at 1 × 10^5^ cells/well in 12-well culture plates and cultured for 1 day in DMEM supplemented with 10% FBS. To inhibit glycogen exocytosis, Pompe fibroblasts were further cultured in the presence of 1 nM colchicine (Sigma-Aldrich) for 5 days, followed by the culture in five different conditions for 2 days; (condition 1) in the presence of 1 nM colchicine, (condition 2) without both colchicine and enzyme treatment (exocytosed), (condition 3) in the presence of 100 nM rGAA, (condition 4) in the presence of 100 nM M6PgP-conjugated rGAA, and (condition 5) in the presence of 100 nM M6PgP-conjugated rGAA with 5 mM M6P. The cells were stained by using PAS staining kit (Merck KGaA, Darmstadt, Germany) according to the manufacturer’s instruction and the resulting images were obtained by light microscopy. The images obtained from each well of 12-well culture plates were analyzed by using NIH Image J software (http://rsb.info.nih.gov/ij/). Strong purple spots over arbitrary threshold values were identified (Supplementary Fig. S6), and their spot areas were quantified by using Image J software. The relative fold changes were obtained by comparing the strong purple spot areas of corresponding cells with those obtained from control cells. Statistical analyses including one-way ANOVA with Tukey’s multiple comparison test were performed by using GrphPad Prism (GraphPad Software, San Diego, CA; http://www.graphpad.com)

## Electronic supplementary material

Supplementary Information
