# Supplementary material for: Lysosomal Targeting Enhancement by Conjugation of Glycopeptides Containing Mannose-6-phosphate Glycans Derived from Glyco-engineered Yeast
Source: Sci Rep. 2018 Jun 7;8:8730. doi: 10.1038/s41598-018-26913-4 (PMC5992200; doi:10.1038/s41598-018-26913-4)
Supplement: Supplementary file 1 — Supplementary Information [file 41598_2018_26913_MOESM1_ESM.pdf]

Supplementary Information

Lysosomal Targeting Enhancement by Conjugation of Glycopeptides Containing Mannose-6-phosphate Glycans Derived from Glyco-engineered Yeast

Ji-Yeon Kang[1, #], Keun Koo Shin[1, #], Ha Hyung Kim[2], Jeong-Ki Min[3,4], Eun Sun Ji[5], Jin Young Kim[5], Ohsuk Kwon[1,6,*], and Doo-Byoung Oh[1,6,*]

[1]Synthetic Biology and Bioengineering Research Center, Korea Research Institute of Bioscience and Biotechnology (KRIBB), Daejeon 34141, Korea. [2]Biotherapeutics and Glycomics Laboratory, College of Pharmacy, Chung-Ang University, Seoul 06944, Korea. [3]Biotherapeutics Translational Research Center, Korea Research Institute of Bioscience and Biotechnology (KRIBB), Daejeon 34141, Korea. [4]Department of Biomolecular Science, University of Science and Technology (UST), Daejeon 34113, Korea. [5]Biomedical Omics Research Center, Korea Basic Science Institute, Ochang, Chungbuk 28119, Korea. [6]Department of Biosystems and Bioengineering, University of Science and Technology (UST), Daejeon 34113, Korea.

Contents

Figure S1. Schematic representation of α(1,2)-mannosidase digestion of P-$Man_8GlcNAc_2$ glycan.

Figure S2. Extracted compound chromatogram of the glycopeptides.

Figure S3. The peptide molecular weight distribution of 23 identified glycopeptides.

Figure S4. The representative CID and HCD MS/MS spectra of the selected glycopeptide.

Figure S5. Analysis of *N*-glycans obtained from rGAA and M6PgP-conjugated rGAA.

Figure S6. PAS-stained Pompe fibroblasts in 12-well culture plate analyzed by image software.

Figure S7. Analysis of *N*-glycans obtained from M6PgP and DBCO-M6PgP.

Figure S8. Amino acid sequences of Dom9-3xFlag-His_8.

Table S1. List of the O-mannosyl glycopeptides identified by HCD and CID MS/MS spectra.

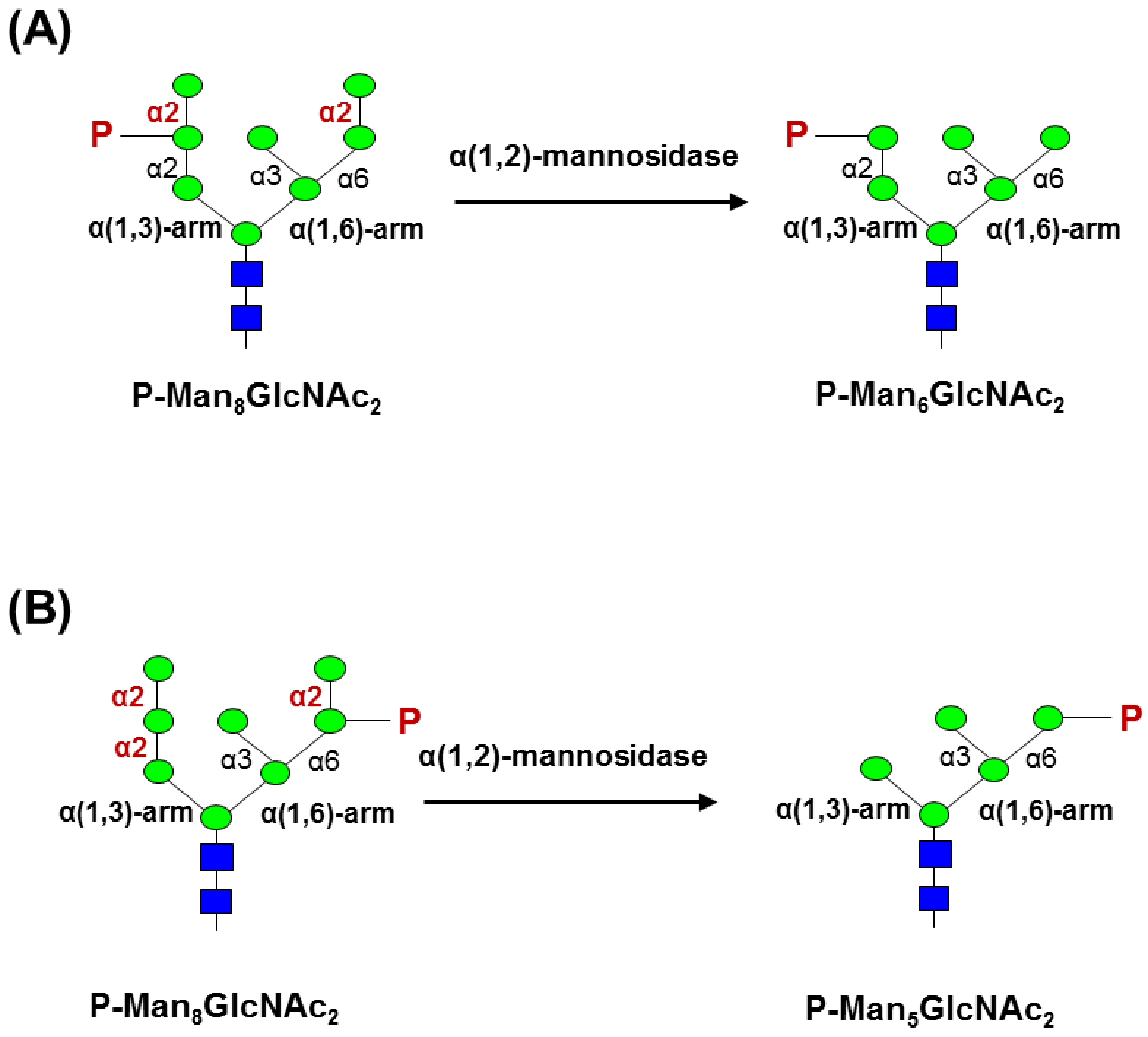

Figure S1. Schematic representation of α(1,2)-mannosidase digestion of P-Man$_8$GlcNAc$_2$ glycan.

The P-Man$_8$GlcNAc$_2$ glycan may have one phosphorylation site at either the penultimate α(1,2)-mannose residue of α(1,3)-arm (A) or the penultimate α(1,6)-mannose residue of α(1,6)-arm (B) because mannosylphosphorylation requires the presence of at least one Man-α(1,2)-Man structure (Wang *et al.*, 1997, *J Biol Chem*). (A) If the phosphorylation site is located on the α(1,3)-arm, P-Man$_6$GlcNAc$_2$ glycan will be created because α(1,2)-mannosidase does not digest the phosphorylated α(1,2)-mannose residue. (B) If the phosphorylation site is located on the α(1,6)-arm, P-Man$_5$GlcNAc$_2$ glycan will be created. The mannose residues which can be removed by α(1,2)-mannosidase were indicated by the red character (α2). Monosaccharide symbols follow the SNFG (Symbol Nomenclature for Glycans) system, details of which are found at the National Center for Biotechnology information (NCBI): green circle, mannose; blue square, GlcNAc; P, phosphate.

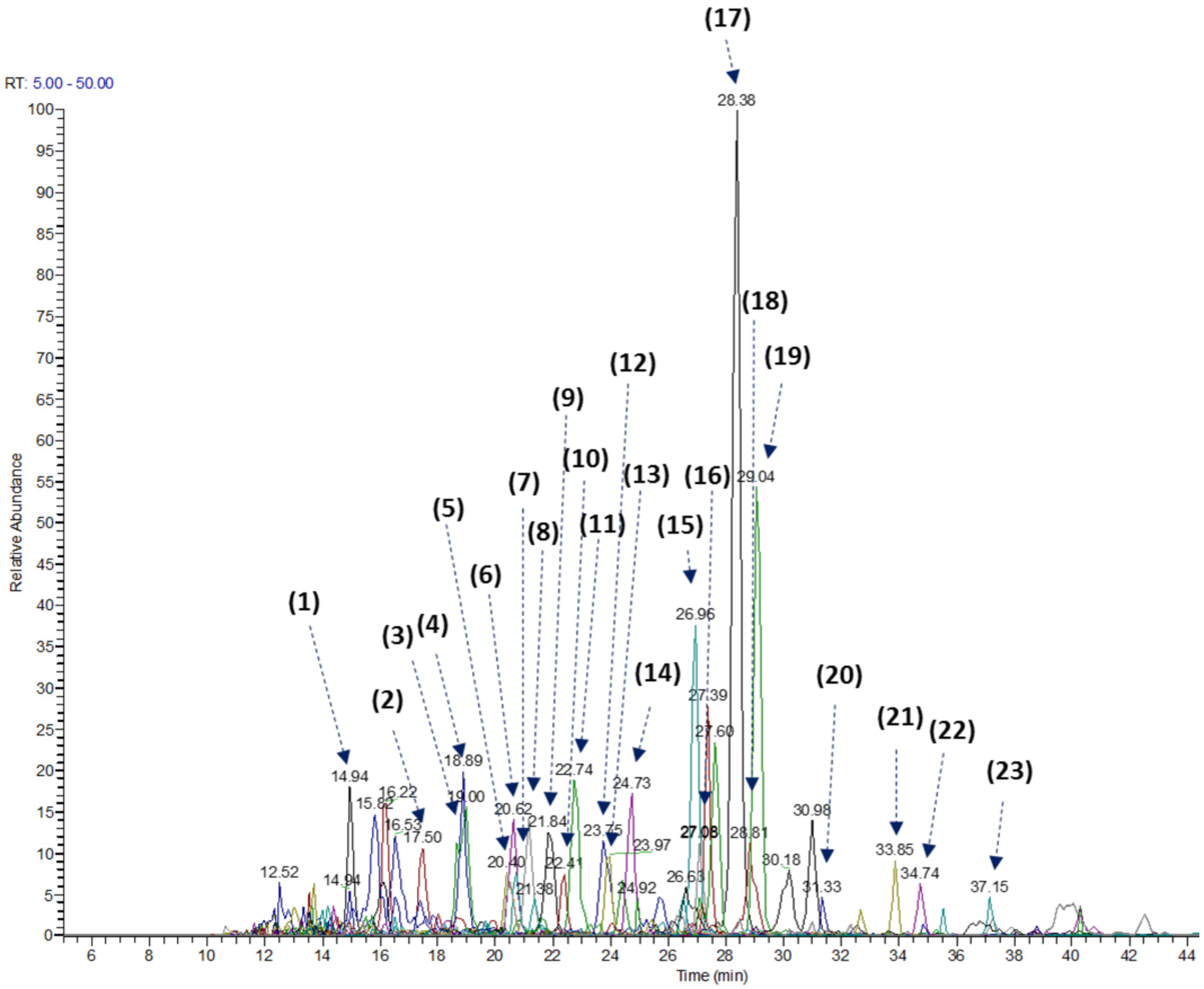

Figure S2. Extracted compound chromatogram of the glycopeptides.

The numbers indicate the glycopeptide peaks identified by collision-induced dissociation (CID) and high energy collision dissociation (HCD) tandem mass (MS/MS) spectra.

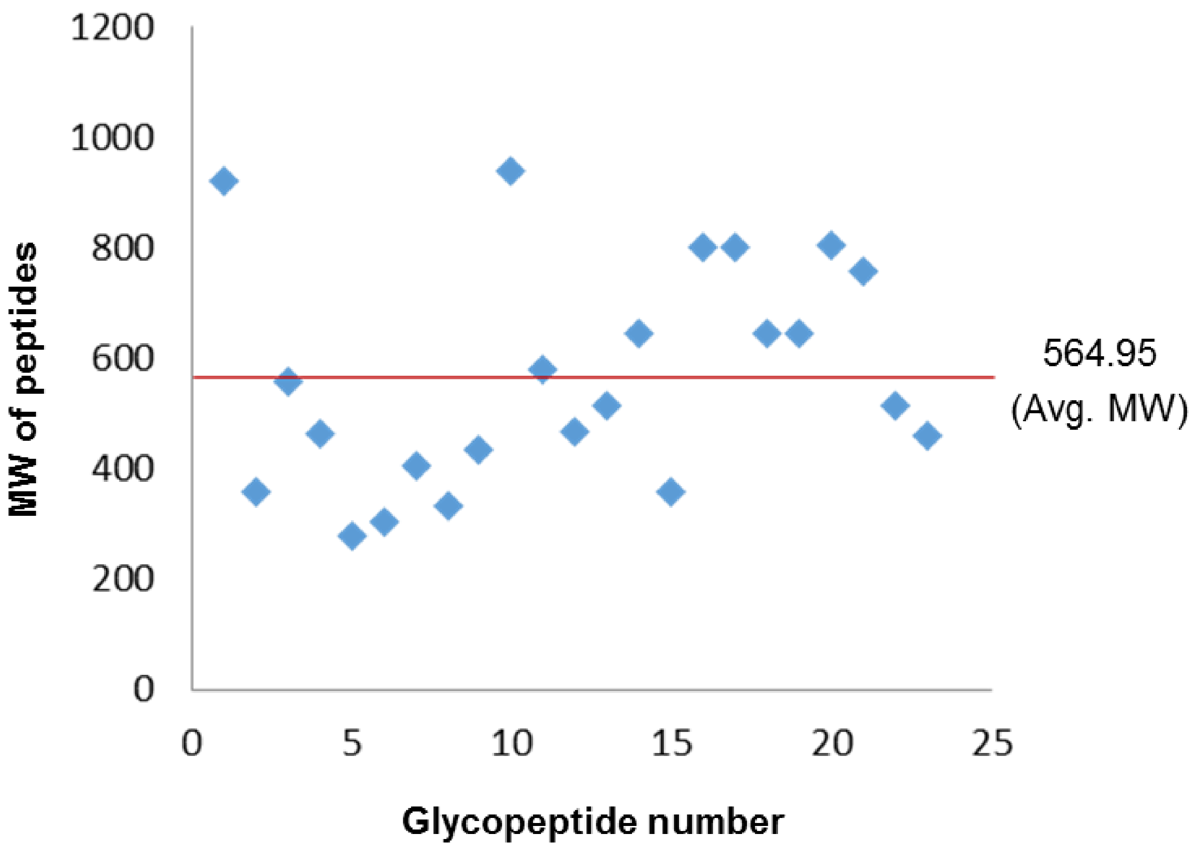

Figure S3. The peptide molecular weight distribution of 23 identified glycopeptides.

The glycopeptide numbers in X-axis correspond to the peak numbers in extracted compound chromatogram in Fig. S2. Their peptide molecular weights (MWs) are represented in the Y-axis.

Peak No.1, m/z = 1392.54 (2+), RT=14.94

MS spectra
m/z 1392.54 (M.W = 2783.08)

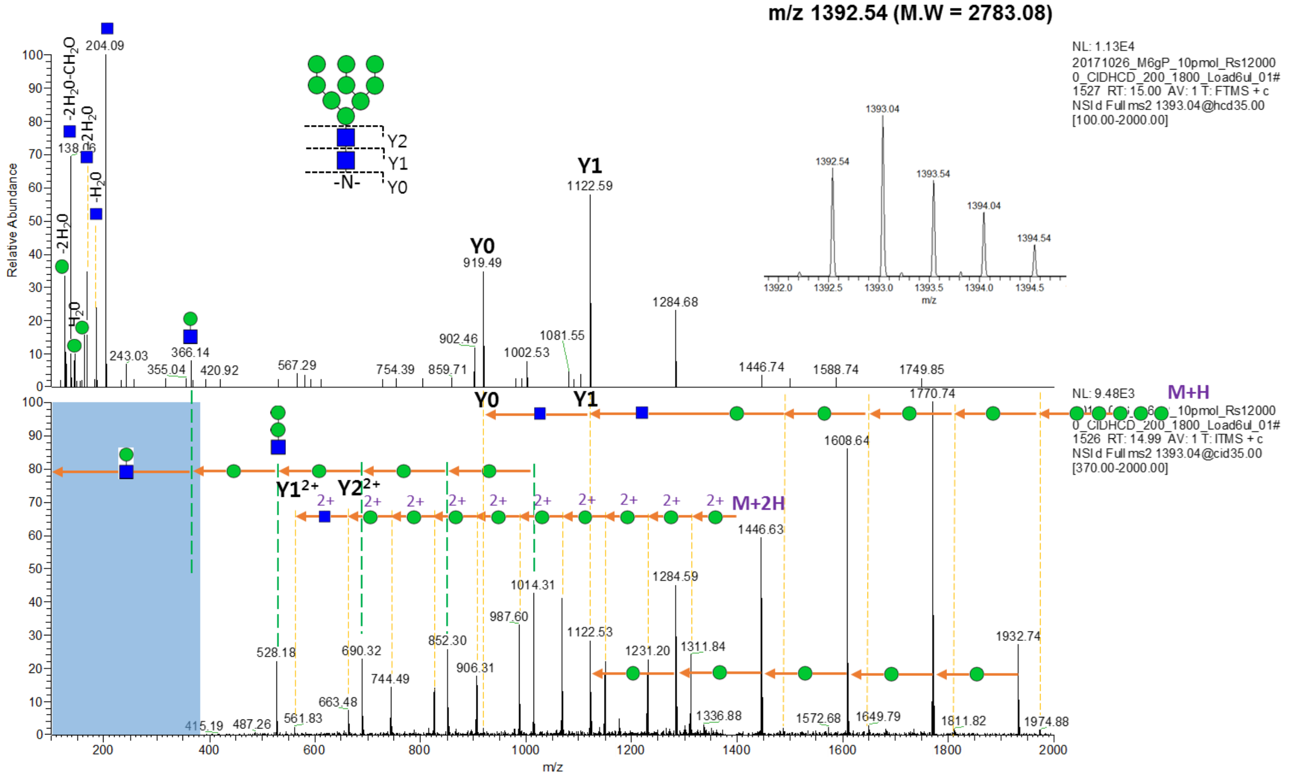

Peak No.4, m/z = 839.825 (2+), RT = 18.89 min

MS spectra
m/z 839.825 (M.W = 1677.65)

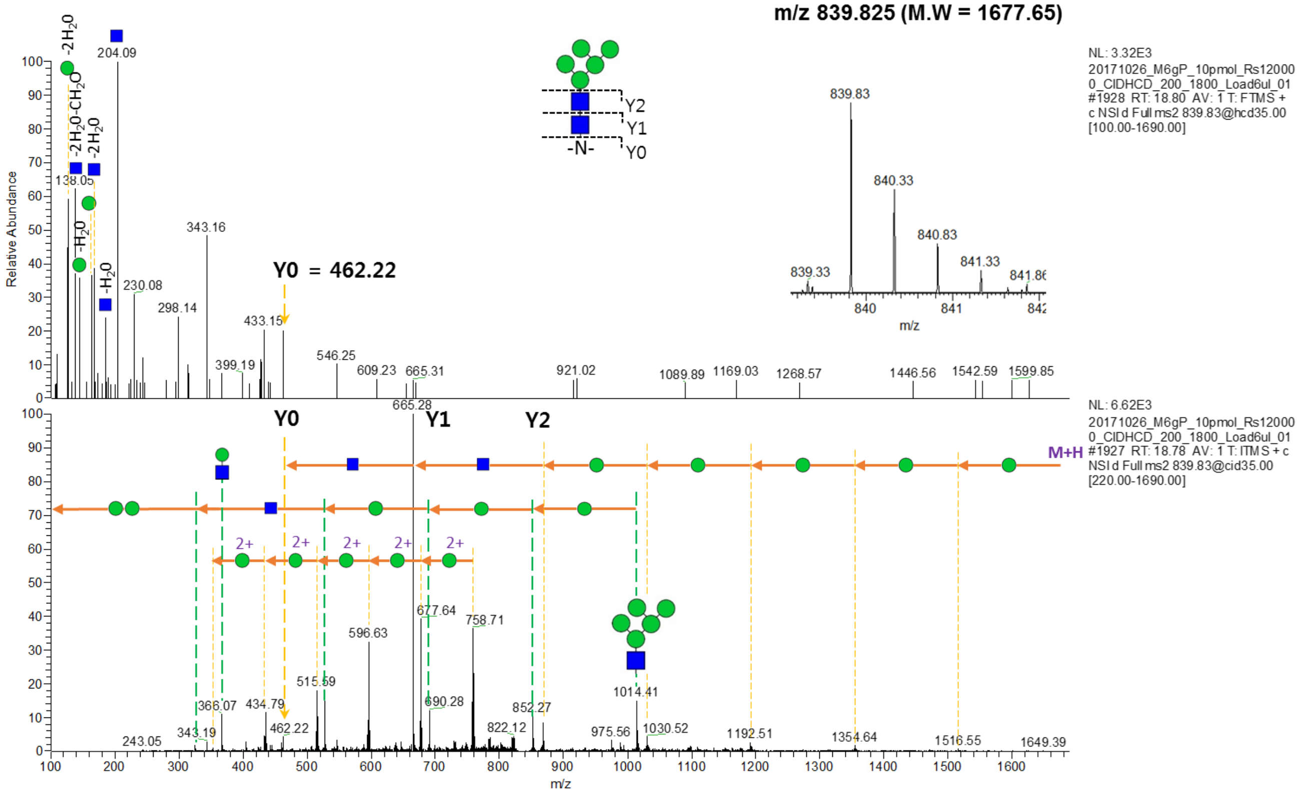

Peak No.5, m/z = 788.76 (2+), RT = 20.4

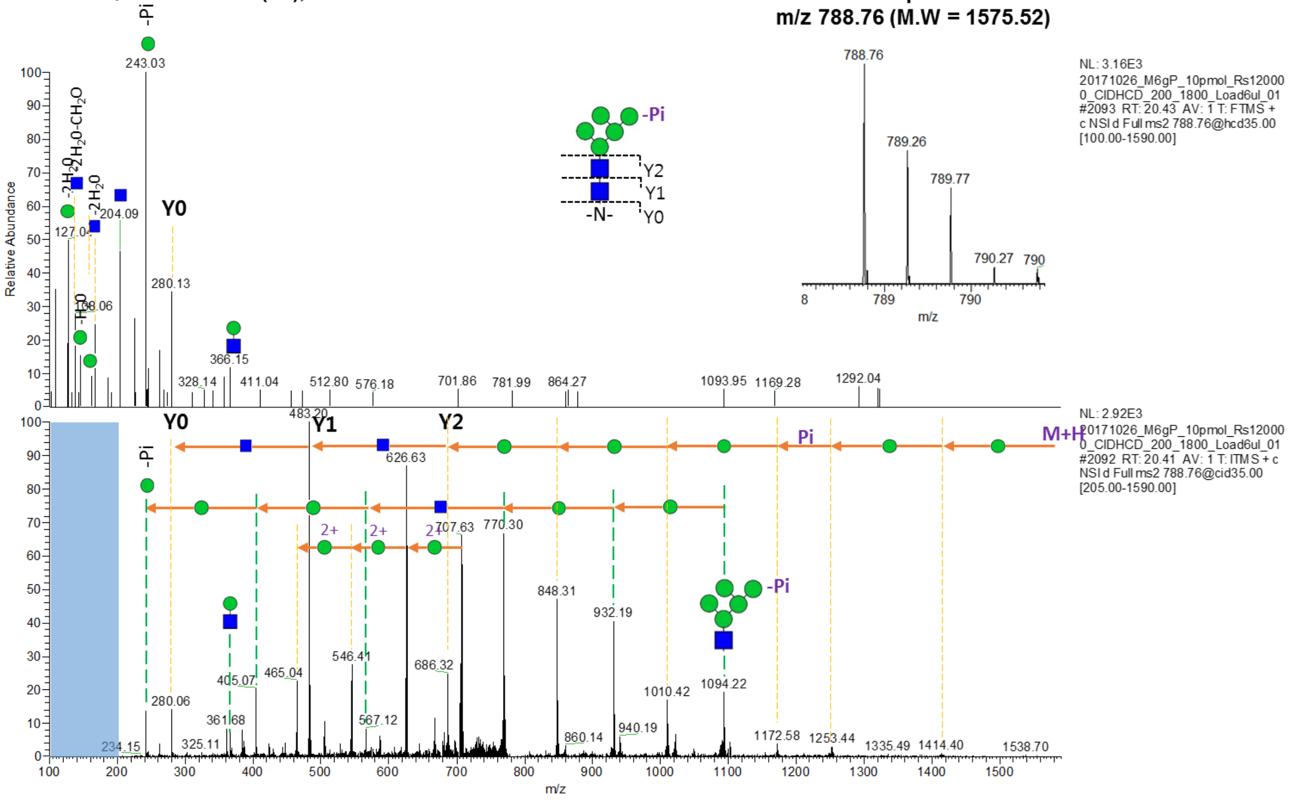

Peak No.6, m/z = 800.28 (2+), RT=20.62

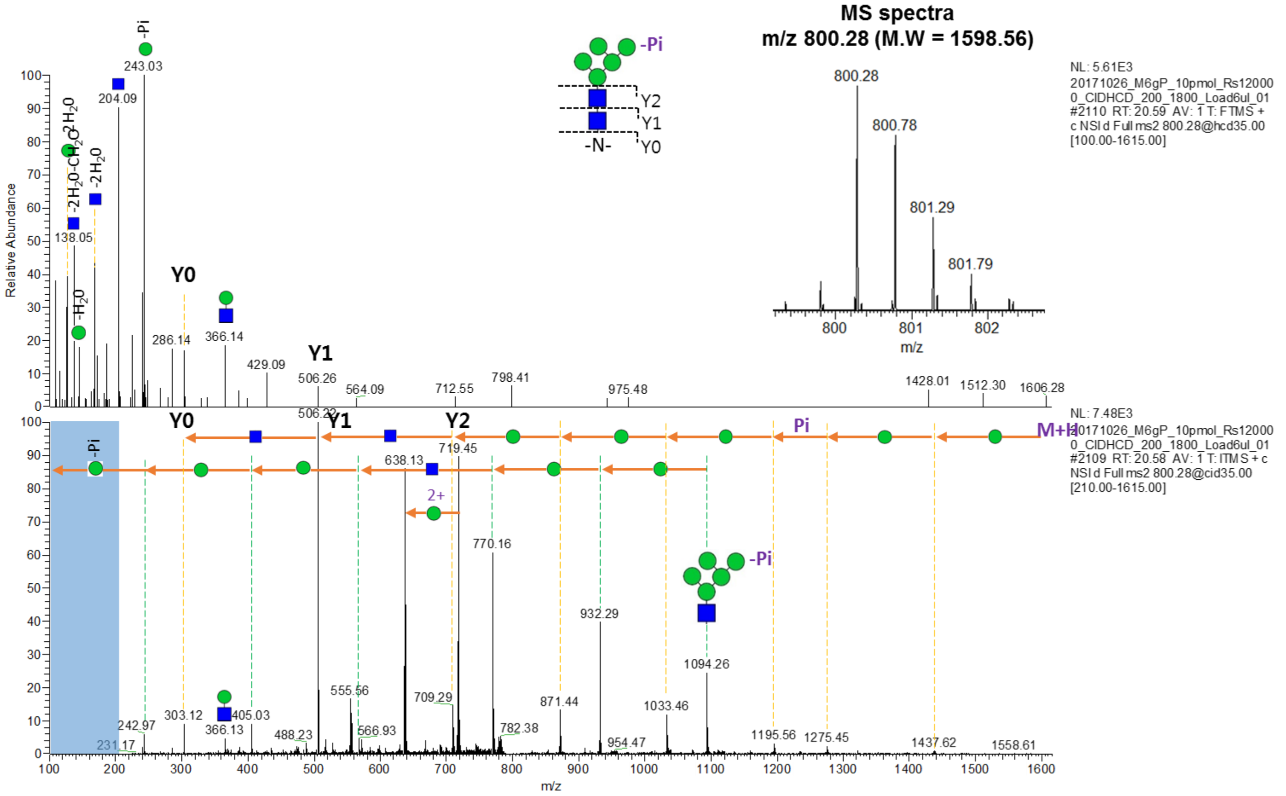

Peak No.10, m/z = 1442.04 (2+), RT=22.41

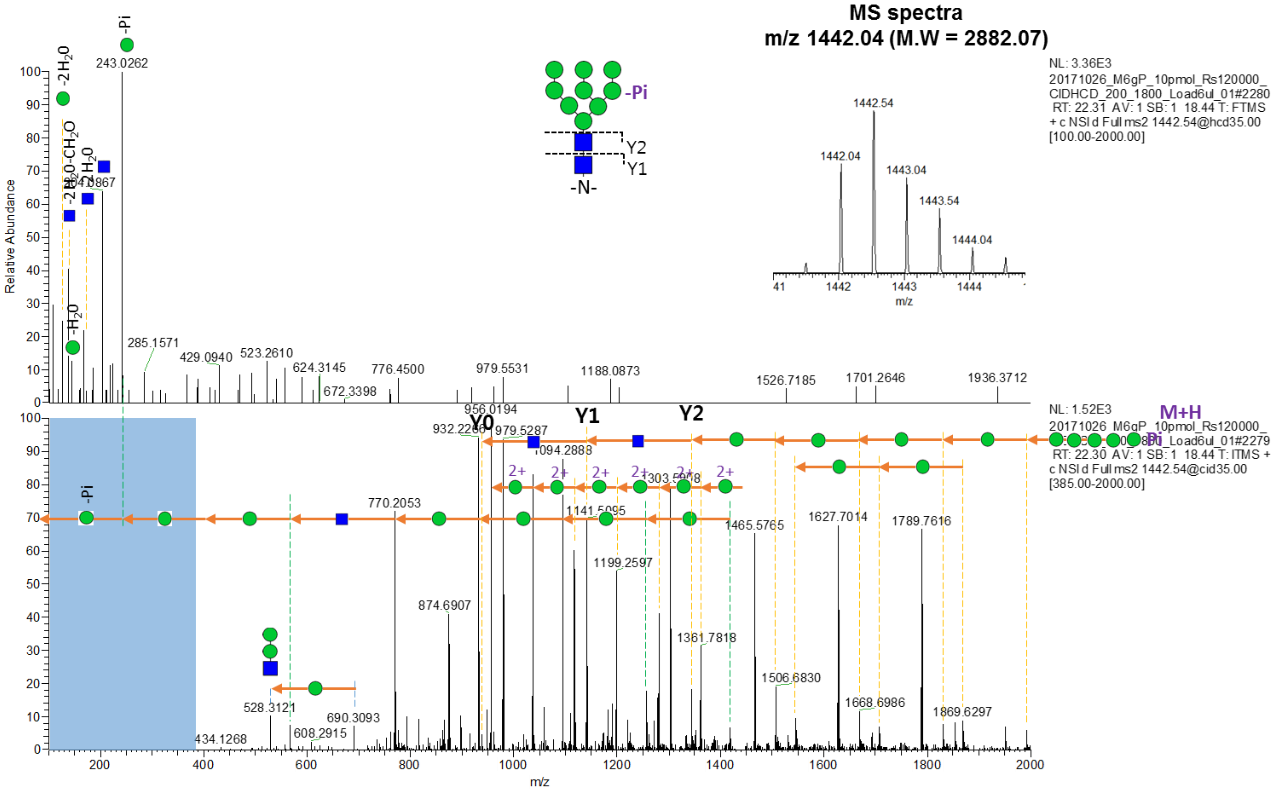

Peak No.11, m/z = 899.33 (2+), RT=22.74

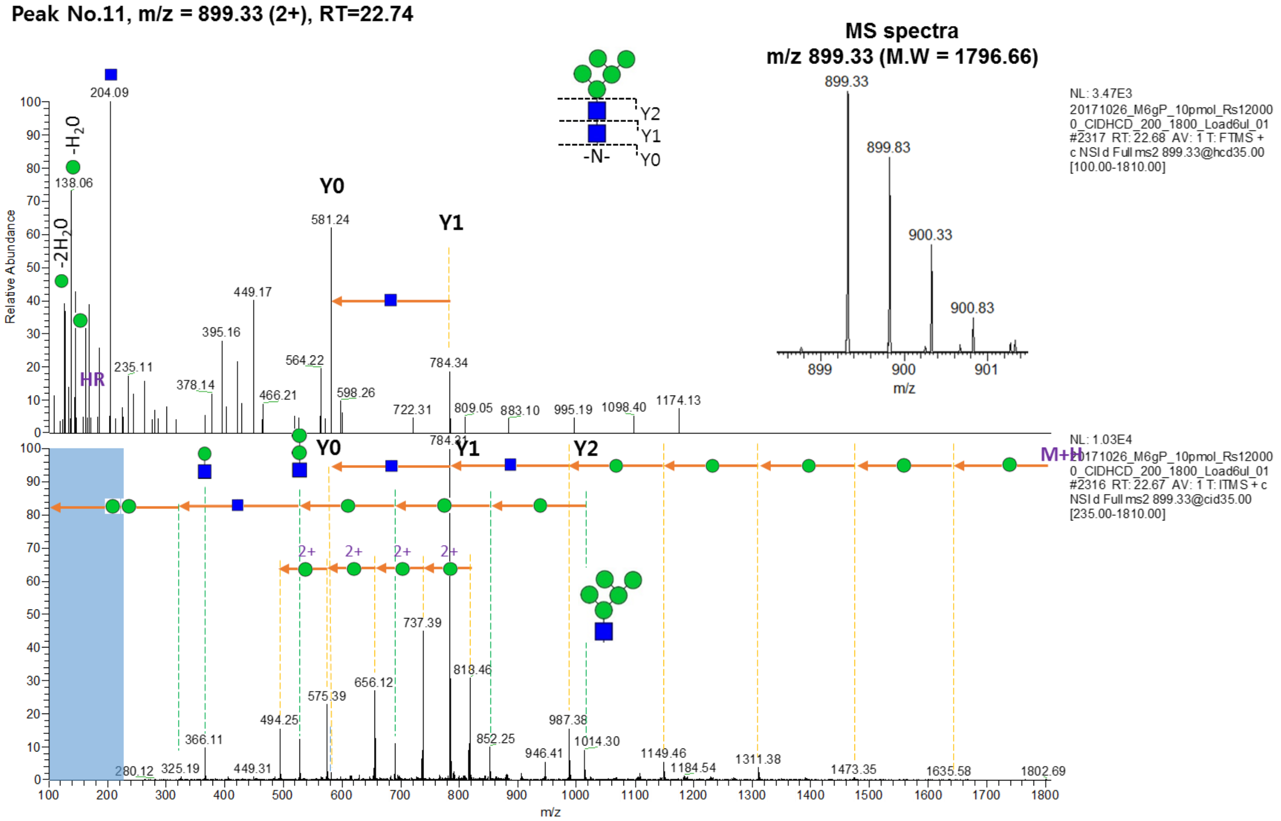

Peak No.14, m/z = 971.36 (2+), RT=24.73

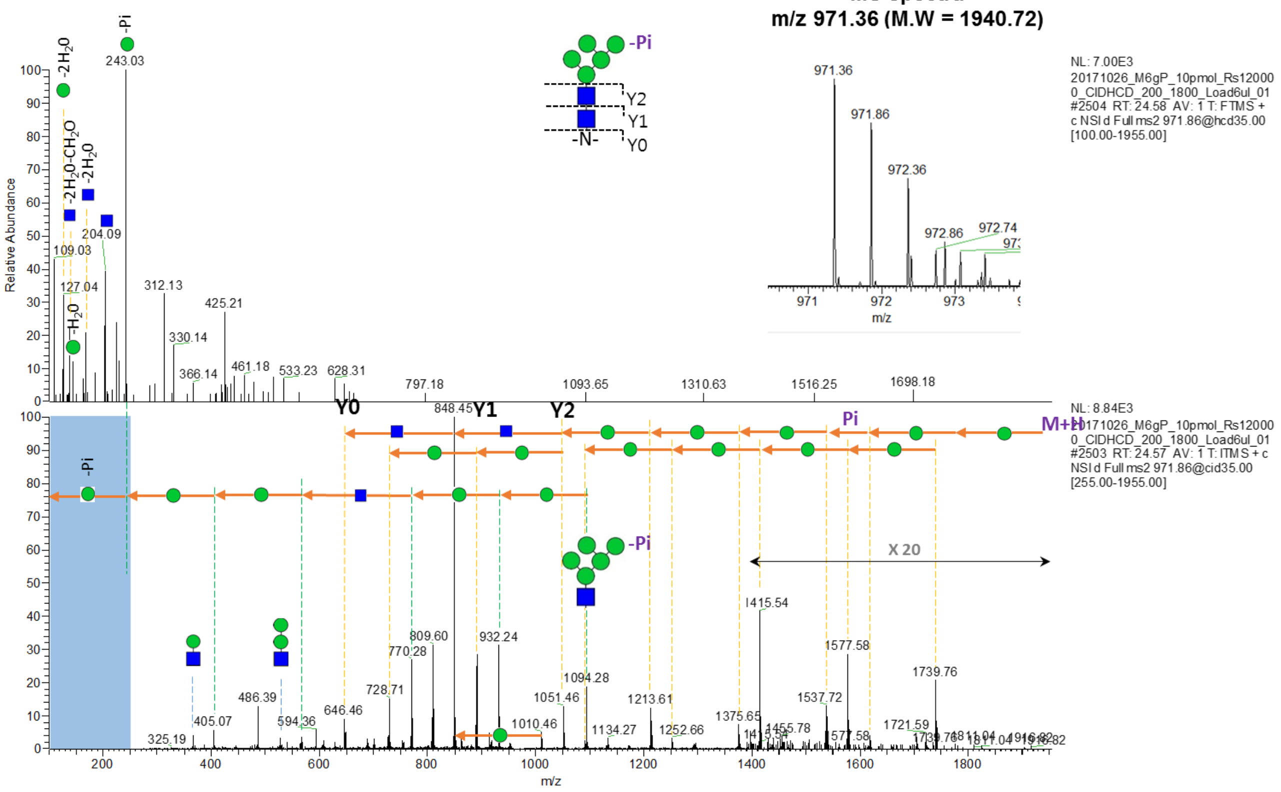

Peak No.17, m/z = 1129.88 (2+), RT=28.38

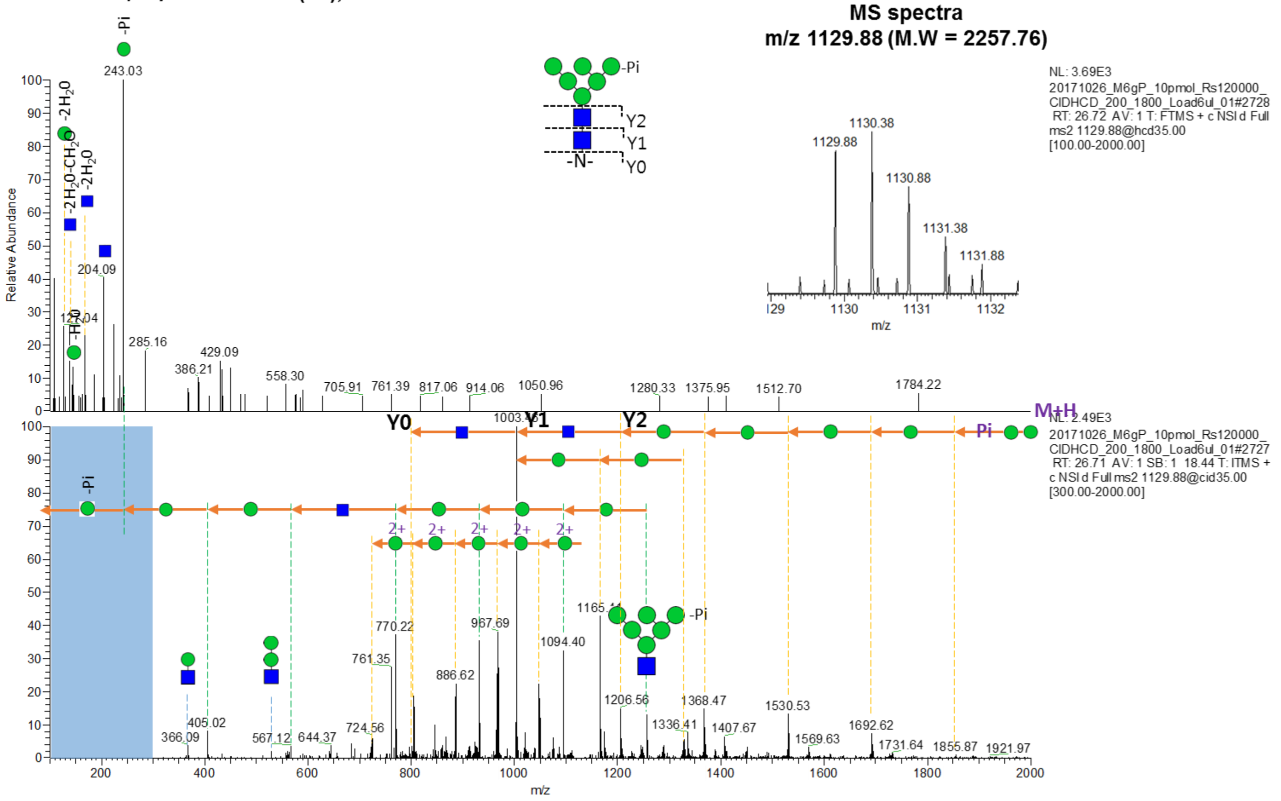

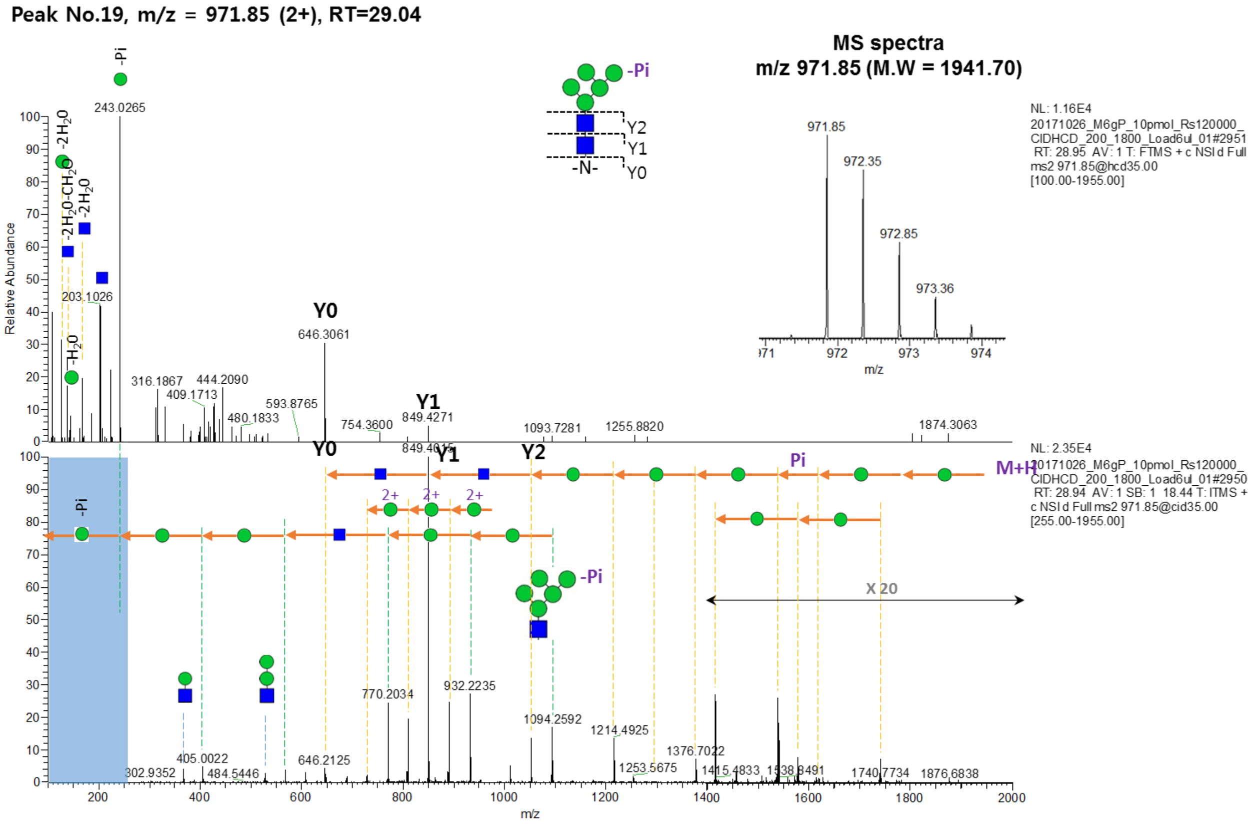

Figure S4. The representative CID and HCD MS/MS spectra of the selected glycopeptide.

The HCD (upper) and CID (lower) spectra of 1, 4, 5, 6, 10, 11, 14, 17, and 19 glycopeptides are represented. Precursor ions observed in high-resolution MS spectra were inserted in right upper part of the HCD spectra. Monosaccharide symbols follow the SNFG (Symbol Nomenclature for Glycans) system, details of which are found at the National Center for Biotechnology Information (NCBI): green circle, mannose; blue square, GlcNAc; P, phosphate.

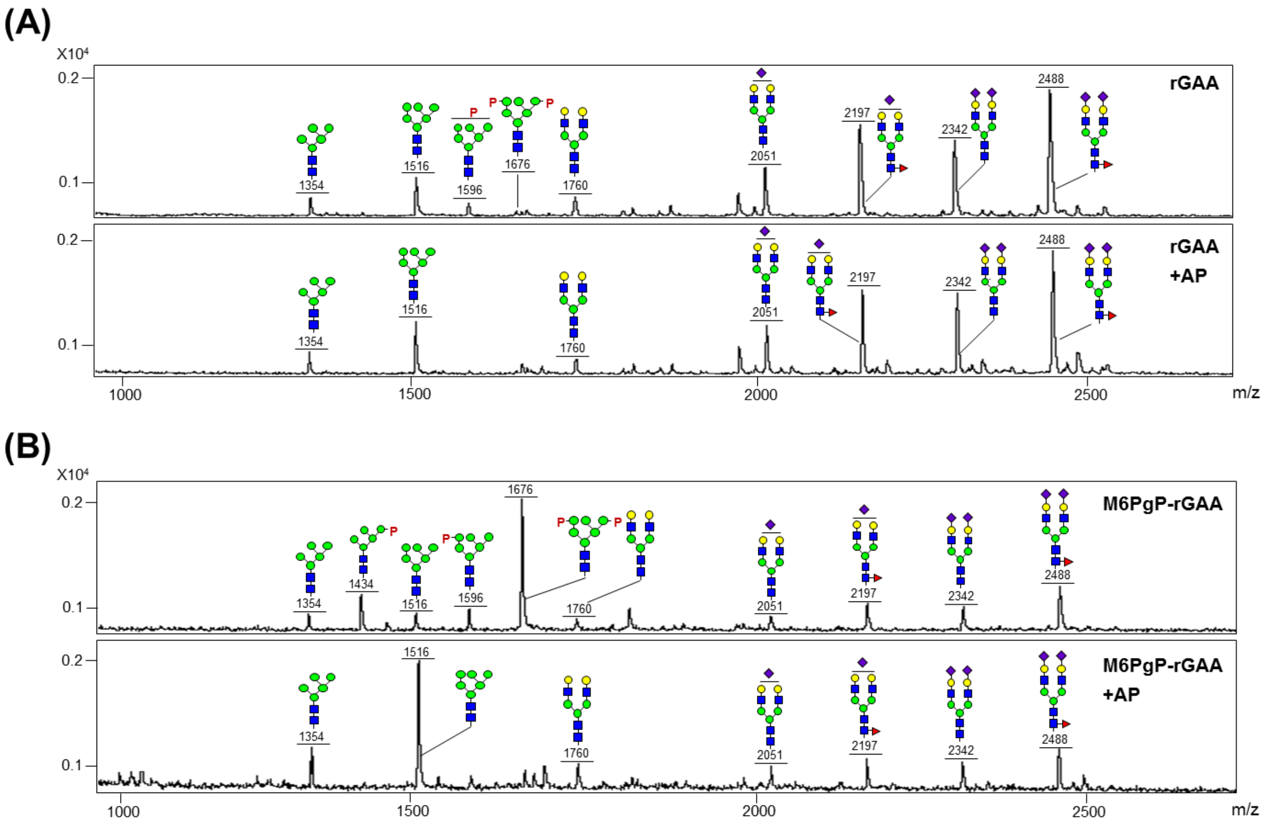

Figure S5. Analysis of *N*-glycans obtained from rGAA and M6PgP-conjugated rGAA.

N-Glycans of rGAA (A) and rGAA-azDB-M6PgP (M6PgP-rGAA) (B) were analyzed by using MALDI-TOF mass spectrometry. After 2-AA labeling, the masses were analyzed in linear negative mode. Alkaline phosphatase was treated to confirm M6P glycans containing exposed phosphate group (+AP). Symbols for glycans are identical to those used in Fig. S1.

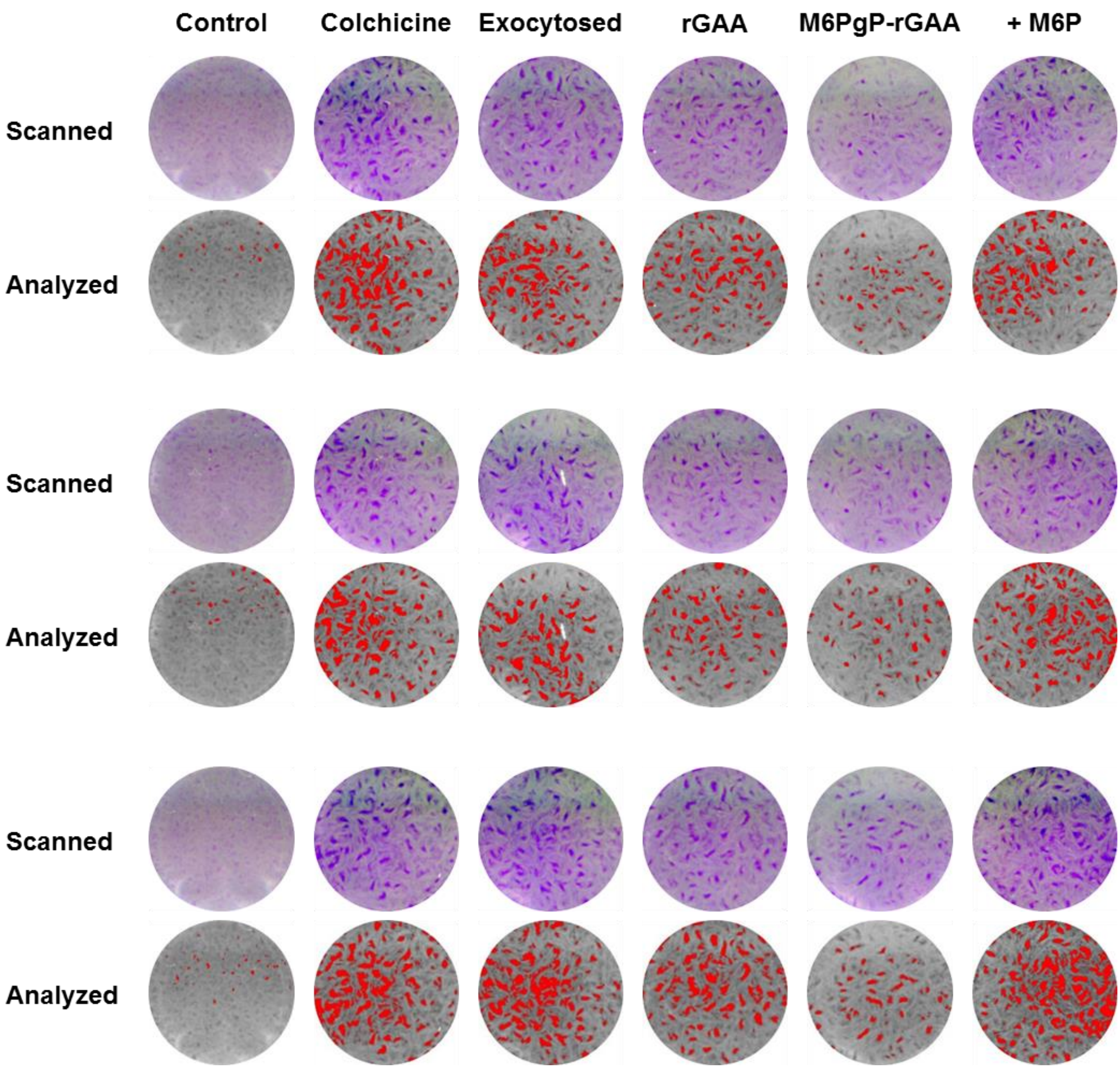

Figure S6. PAS-stained Pompe fibroblasts in 12-well culture plate analyzed by image software. Glycogens in Pompe fibroblasts cultured in the presence of colchicine were strongly PAS-stained whereas control cells were rarely stained. The accumulated glycogens in the cells were spontaneously exocytosed or digested by addition of rGAA or M6PgP-conjugated rGAA (M6PgP-rGAA). Free M6Ps competitively inhibited glycogen clearance (+ M6P). The images obtained from wells of culture plate (Scanned) were analyzed by using NIH Image J software (http://rsb.info.nih.gov/ij/). Strong purple spots over arbitrary threshold values were represented with red color (Analyzed), which were used for quantification. Three independent experimental results are shown.

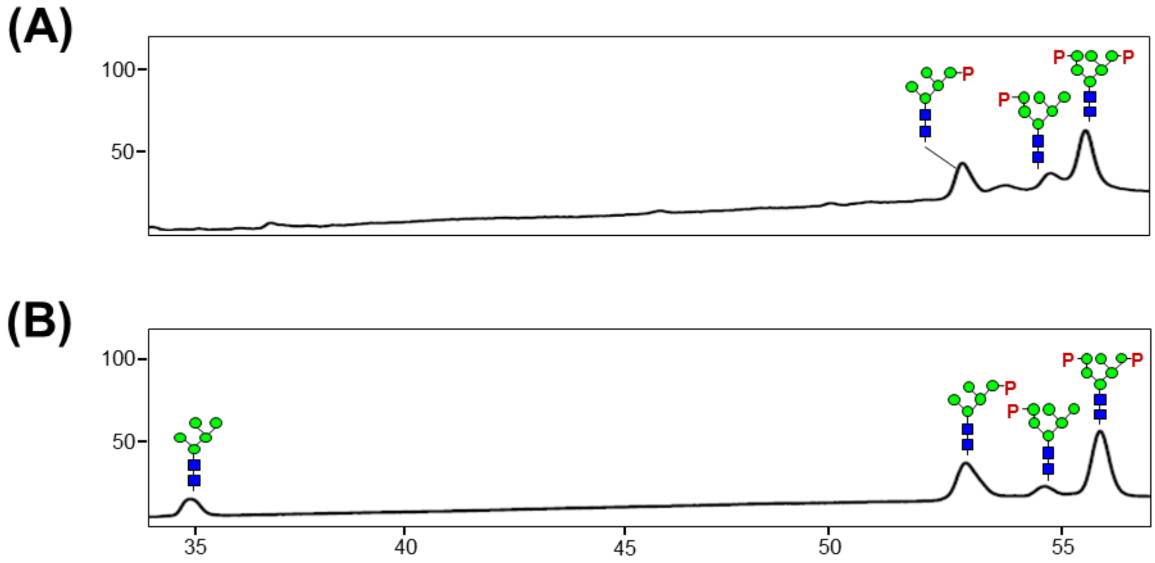

Figure S7. Analysis of *N*-glycans obtained from M6PgP and DBCO-M6PgP.

N-Glycans of M6PgP (A) and DBCO-M6PgP (B) were analyzed by using HPLC after 2-AA labeling. The peaks were identified by determination of the masses of collected peaks. Symbols for glycans are identical to those used in Fig. S1.

```
mrfpsiftav lfaassalaa pvntttedet aqipaeavig yldlegdfdv
avlpfsnstn ngllfintti asiaakeegv sldkreaeaw hwlqlkpgqp
mykreaeaVV RAEGDYCEVR DPRHGNLYNL IPLGLNDTVV RAGEYTYYFR
VCGELTSGVC PTSDKSKVIS SCQEKRGPQG FQKVAGLFNQ KLTYENGVLK
MNYTGGDTCH KVYQRSTTIF FYCDRSTQAP VFLQETSDCS YLFEWRTQYA
```

CP**DYKDHDGD YKDHDIDYKD DDDK**<u>HHHHHH HH</u>

Figure S8. Amino acid sequences of Dom9-3xFlag-His$_8$.

Sequences of yeast mating factor α leader and domain 9 of the cation-independent M6P receptor (Dom9) are represented by lower and upper cases, respectively. Three tandem Flag-tag and His$_8$-tag added to C-terminal of Dom9 are shown in bold and underlined characters respectively.

Table S1. List of the *O*-mannosyl glycopeptides identified by HCD and CID MS/MS spectra.

Peak No	RT (min)	m/z	Charge State	Experimental MW	Identified glycan	MW of glycan	Y0 ion*	Y1 ion*	Calculated MW
1	12.06	938.42	2	1874.84	Hex(6) †	972.32	903.56	1065.64	1874.82
2	11.98	1019.45	2	2036.89	Hex(7)	1134.37	903.56	1065.64	2036.88
3	13.00	1059.43	2	2116.86	Hex(7)P	1214.37	903.57	1065.64	2116.84
4	15.82	802.33	2	1602.67	Hex(5)	810.27	793.52	955.40	1602.65
5	15.21	883.36	2	1764.72	Hex(6)	972.32	793.46	955.40	1764.70
6	14.09	964.36	2	1926.77	Hex(7)	1134.37	793.43	955.16	1926.76

*MS/MS spectra delivers information about the sugar structure *via* glycosidic bond cleavages, and provides molecular mass information for the modified peptide in the form of Y0 (peptide + H^+) and Y1 (peptide + Hex(1) + H^+) ions.

† Abbreviations : Hexose (Hex), Phosphate (P)
